# Overlapping Yet Response-Specific Transcriptome Alterations Characterize the Nature of Tobacco–*Pseudomonas syringae* Interactions

**DOI:** 10.3389/fpls.2016.00251

**Published:** 2016-03-07

**Authors:** Zoltán Bozsó, Péter G. Ott, Evelin Kámán-Tóth, Gábor F. Bognár, Miklós Pogány, Ágnes Szatmári

**Affiliations:** Department of Pathophysiology, Centre for Agricultural Research, Plant Protection Institute, Hungarian Academy of SciencesBudapest, Hungary

**Keywords:** pattern triggered immunity (PTI), effector triggered immunity (ETI), compatible interaction, transcriptome, tobacco, *Pseudomonas syringae*, signal transduction

## Abstract

In this study transcriptomic alterations of bacterially induced pattern triggered immunity (PTI) were compared with other types of tobacco–*Pseudomonas* interactions. In addition, using pharmacological agents we blocked some signal transduction pathways (Ca^2+^ influx, kinases, phospholipases, proteasomic protein degradation) to find out how they contribute to gene expression during PTI. PTI is the first defense response of plant cells to microbes, elicited by their widely conserved molecular patterns. Tobacco is an important model of *Solanaceae* to study resistance responses, including defense mechanisms against bacteria. In spite of these facts the transcription regulation of tobacco genes during different types of plant bacterial interactions is not well-described. In this paper we compared the tobacco transcriptomic alterations in microarray experiments induced by (i) PTI inducer *Pseudomonas syringae* pv. *syringae* type III secretion mutant (*hrcC*) at earlier (6 h post inoculation) and later (48 hpi) stages of defense, (ii) wild type *P. syringae* (6 hpi) that causes effector triggered immunity (ETI) and cell death (HR), and (iii) disease-causing *P. syringae* pv. *tabaci* (6 hpi). Among the different treatments the highest overlap was between the PTI and ETI at 6 hpi, however, there were groups of genes with specifically altered activity for either type of defenses. Instead of quantitative effects of the virulent *P. tabaci* on PTI-related genes it influenced transcription qualitatively and blocked the expression changes of a special set of genes including ones involved in signal transduction and transcription regulation. *P. tabaci* specifically activated or repressed other groups of genes seemingly not related to either PTI or ETI. Kinase and phospholipase A inhibitors had highest impacts on the PTI response and effects of these signal inhibitors on transcription greatly overlapped. Remarkable interactions of phospholipase C-related pathways with the proteasomal system were also observable. Genes specifically affected by virulent *P. tabaci* belonged to various previously identified signaling routes, suggesting that compatible pathogens may modulate diverse signaling pathways of PTI to overcome plant defense.

## Introduction

Survival of plants is dependent on cellular recognition of microorganisms and a quick induction of defense responses. Plants possess different layers of resistance mechanisms to restrict multiplication of microbes in their tissues. Pathogenic, non-host pathogens, and saprophytic bacteria may enter the intercellular space of leaves passively e.g., via rain drops, or actively using their motile organelles on a leaf surface. The first layer of defense response activated in plant cells is triggered by microbe- or pathogen-associated molecular pattern(s) (MAMPs/PAMPs) of microorganisms (Mackey and McFall, 2006; Newman et al., 2013). MAMPs are common within groups of microbes (e.g., fungi or bacteria) irrespective of their life style i.e., pathogen or saprophytic. MAMPs can be surface secreted or intracellular bacterial molecules e.g., flagellin, lipopolysaccharide, peptidoglycan, cold shock protein, elongation factor Tu, or superoxide dismutase (Dow et al., 2000; Felix and Boller, 2003; Kunze et al., 2004; Watt et al., 2006; Gust et al., 2007; Boller and Felix, 2009). The MAMP-induced resistance response is called pattern triggered immunity (PTI), also known as basal resistance. Generally, PTI is a symptomless plant response accompanied by several signaling, transcriptional, biochemical and structural changes (Nicaise et al., 2009). In compatible plant–pathogen interactions when bacteria are able to multiply the pathogen successfully blocks PTI (Jakobek et al., 1993; Hauck et al., 2003; Klement et al., 2003; Keshavarzi et al., 2004; Boller and He, 2009).

The next layer of plant defense is activated by the recognition of effector protein(s) which are injected into the host cell by a type III secretion system (T3SS). This process is known as effector-triggered immunity (ETI; Jones and Dangl, 2006). Effectors are recognized directly or indirectly by NB-LRR type resistance proteins (Gassmann and Bhattacharjee, 2012). ETI can be accompanied by plant cell death that is known as hypersensitive reaction (HR). In contrast to PTI, ETI is typically induced by living, metabolically active bacteria (Klement, 1963, 1982).

Whole-genome transcriptomic studies of *Arabidopsis* and other plant species showed that during both PTI and ETI high number of genes were up- or down regulated soon after elicitation. These studies also implicated that there is a significant overlap between the expression profiles of various plant species during PTI or ETI (e.g., Tao et al., 2003; Navarro et al., 2004; Bozsó et al., 2009). It was also shown that a considerable part of the differences was quantitative. The amplitude of the response is usually highest during ETI which may reflect to more prolonged and robust response than in PTI. Recent results further support that ETI and PTI use common regulatory networks, since the loss of four main regulating sectors (salicylate, jasmonate, ethylene, and phytoalexin-deficient 4) may decrease the effectiveness of both PTI and ETI ~80% (Tsuda et al., 2009). It is also established that during compatible interactions virulence factors (e.g., T3SS effectors or toxins) of the pathogen may inhibit the transcription of several defense associated genes activated during PTI and/or ETI (Thilmony et al., 2006; Truman et al., 2006; Rosli et al., 2013). This phenomenon is also known as effector-triggered susceptibility (ETS), since effector activities in compatible interactions on host targets are involved in the establishment of susceptible interactions (Jones and Dangl, 2006).

Several elements of PTI-related signal transduction pathways have been described. The results imply that these signaling mechanisms constitute a network rather than a single linear pathway. The identified receptors of PTI elicitors are cell membrane embedded LRR-receptor kinases (Boller and Felix, 2009). In case of flagellin perception ligand binding induces the association of different RLKs and receptor-like cytoplasmic kinases (RLCKs) together with phosphorylation and transphosphorylation events. This leads to the activation of a MAP kinase cascade (Asai et al., 2002; Pitzschke et al., 2009; Tena et al., 2011). Another important signal transduction event during PTI activation is calcium influx. The sources of the Ca^2+^ increase can be extracellular and/or intracellular (e.g., endoplasmic reticulum or vacuole). Calcium channels are phosphorylated and Ca^2+^ influx activates calcium-dependent protein kinases (CDPKs). CDPKs and MAP kinases regulate transcription factors separately or in cooperation (Boudsocq et al., 2010; Boudsocq and Sheen, 2013). Calcium binding proteins such as calmodulin (CAM) or calcineurin B-like proteins (CBLs) together with CDPKs transmit and amplify the signal (Batistič and Kudla, 2012).

Lipids are not only structural constituents of cells but they are also important signaling molecules. Production of lipid derived signals is regulated by enzymes including phospholipase A, C, or D. Phospholipase A (PLA) enzymes hydrolyze phospholipids at sn-1 and/or sn-2 positions and produce free fatty acids (FFAs) and lysophospholipids (Canonne et al., 2011). FFAs can function as a second messenger or as a precursor of oxylipins (Munnik and Testerink, 2009). Lysophospholipids may also have a second messenger function, e.g., can activate a H^+^/Na^+^ vacuolar antiporter to decrease the intracellular pH and regulate phytoalexin biosynthesis (Viehweger et al., 2002). It has been also observed that PLA_2α_ rapidly translocates to the apoplasts after infiltration of avirulent bacteria (Jung et al., 2012). PLA_2_ (together with PLC and PLD) may also be involved in the regulation of microtubule organization (Gardiner et al., 2008; Pleskot et al., 2014).

In plants both PLC and PLD can produce phosphatidic acid (PA). PLC hydrolyses phosphatidylinositol and its phosphorylated derivative to produce diacylglycerol (DAG) that is phosphorylated to PA by DAG kinase. PLD generates PA directly by hydrolyzing structural phospholipids like phosphatidylcholine (PC) (Canonne et al., 2011). On one hand PA-binding can modify the activity of some protein(s) e.g., kinases and phosphatases (Anthony et al., 2004; Testerink et al., 2004, 2007). On the other hand PA may act as a membrane anchor to link different proteins together. PA can also be a substrate for the production of other lipid signal molecules (Wang, 2004). PA is able to induce reactive oxygen intermediate production and defense gene activation (Sang et al., 2001; Andersson et al., 2006). Activity of PLC also results in the formation of inositol 1,4,5-trisphosphate (IP_3_). IP_3_ may rapidly be converted into IP_6_ and both products can trigger the release of Ca^2+^ from intercellular stores.

Besides phosphorylation, the ubiquitin-mediated proteolysis is one of the most important post-translational mechanisms that control transcription regulators (Geng et al., 2012). During ubiquitination a covalent conjugation of conserved ubiquitin molecules to specific protein substrates is performed which finally leads to the digestion of target proteins in the multi-subunit proteasomes (Geng et al., 2012). The role of proteasomes in PTI begins with a negative-regulation of the amount of MAMP/PAMP receptors (Furlan et al., 2012). For instance, after elicitor treatment the FLS2 flagellin receptor is internalized by endocytosis and degraded by proteasomes. This process is part of a feedback regulation of PTI that controls the intensity and duration of resistance responses (Robatzek et al., 2006; Lu et al., 2011). Receptor associated proteasomic degradation may be active in positive-regulation as well. For example, phosphorylation of XB3 ubiquitin ligase by XA21 receptor-like kinase positively regulates XA21 signaling (Wang et al., 2006). Regulation of transcription factor and transcription initiation complex stability can also be an important function in defense gene regulation through the proteasome system and could be effectively influenced by a pharmacological approach (Spoel et al., 2009; Huang et al., 2013). Defense-related hormone responses such as jasmonate, auxin, and abscisic acid signaling are also proteasome-dependent processes (Santner and Estelle, 2010; Liu and Stone, 2011; Pauwels and Goossens, 2011). The importance of the proteasomal system in plant–microbe interactions is demonstrated by the facts that phytopathogenic bacteria are able to manipulate this system for their survival. Bacteria can use toxin(s) and proteinaceous effectors to inhibit or manipulate the plant proteasomal system (Groll et al., 2008; Marino et al., 2012; Üstün and Börnke, 2014).

In our experiments we have investigated the transcriptomic changes in tobacco leaves during different types of bacterial interactions. ETI was triggered by an incompatible *Pseudomonas syringae* pv. *syringae* bacterium strain that causes HR-type cell death, while PTI was induced by the *hrcC* HR-negative T3SS mutant of this bacterial strain. Moreover, we have also studied how the compatible pathogen, *P. syringae* pv. *tabaci* affects the tobacco transcriptome to turn leaves into a favorable environment for its multiplication. We were looking for common and specific expression patterns characteristic of ETI and PTI and analyzed how expression patterns were affected by a compatible interaction. To gain a more detailed picture about PTI regulation in tobacco we also tested the possible roles of some signal transduction pathways in the transcriptomic response during PTI, by using pharmacological agents.

## Materials and methods

### Plant material

Tobacco plants (*Nicotiana tabacum* L. cv. Samsun) were planted and grown in greenhouse in soil (general potting mix from peat, cow manure and perlite, pH 6–7). Two days before inoculation 6–8-week-old tobacco plants were placed in a growth chamber set to 16/8 h light/dark period at 20°C.

### Bacterial and inhibitor treatments of plant leaves

*Pseudomonas syringae* pv. *syringae* 61 (*P. syringae*) HR-positive wild type strain containing hopA1 (hrmA, HoPsyA) effector that greatly affects HR-inducing ability of this strain (Heu and Hutcheson, 1993; Bozsó et al., 1999), *P. syringae* pv. *syringae* 61 *hrcC* (*P. syringae hrcC*) HR-negative T3SS deficient mutant (61-1530B, Alan Collmer, Cornell University, Ithaca, USA) and virulent *P. syringae* pv. *tabaci* H10 compatible on tobacco plant (NCAIM B.01601, National Collection of Agricultural and Industrial Microorganisms, Budapest, Hungary) were grown overnight at 27°C on King's B medium (King et al., 1954). In case of *P. syringae hrcC* medium was supplemented with 50 μg/ml kanamycin sulfate.

For inoculum preparations bacterial cells were suspended in distilled water and adjusted to 10^8^ CFU/ml density using a spectrophotometer (OD_600_ = 0.21). The inocula were injected into intercellular spaces of middle aged, fully developed tobacco leaves with a hypodermic syringe fitted with 25 gauge needle (Klement, 1990).

For blocking signaling pathways during PTI different inhibitors were mixed with the bacterial suspensions. The final concentrations of inhibitors used to inhibit the different signal transduction pathways were the followings: 1.5 mM LaCl_2_, 50 μM neomycin sulfate, 100 μM aristolochic acid, 1.5 μM K252a, 50 μM MG115 (all chemicals were purchased from Sigma-Aldrich, USA). All inhibitor stocks were 200X concentrated and diluted in bacterial suspensions immediately before leaf infiltration. LaCl_2_ and neomycin sulfate were dissolved in distilled water. Aristolochic acid, K252a and MG115 were dissolved in DMSO. For these latter treatments the control bacterial suspensions were supplemented with DMSO diluted 200X times.

To inactivate bacteria the *P. syringae* pv. *tabaci* suspension was supplemented with 50 μg/ml kanamycin sulfate and incubated for 10 min then centrifuged and washed with distilled water two times. To check effectiveness of the inactivation, kanamycin treated bacterial suspensions were spread on King's B agar plates.

### Sample preparation for microarray experiments

For experiments comparing the effects of different types of bacteria on the transcriptome of tobacco, one half of middle leaves were injected with water as a control and the other half of leaves were infiltrated with different bacterial suspensions. To investigate effects of various signal transduction inhibitors on gene expression during PTI one half of leaves were injected with PTI-inducing *P. syringae hrcC* suspension (supplemented with DMSO when the inhibitor stock had been dissolved in DMSO) and the other half of leaves were injected with *P. syringae hrcC* suspension supplemented with the particular inhibitor.

For RNA preparation ~100 mg leaf tissue was collected from three different plants at indicated times after inoculation, frozen in liquid nitrogen and stored at −70°C. Each experiment was carried out in three independent replicates. Total RNA was extracted from frozen tissues using Qiagen RNeasy Plant Mini kit and Qiagen RNase-free Dnase Set (Qiagen, USA), then re-purified and concentrated by Microcon-30 (Millipore, USA) columns. The concentration and quality of isolated RNA samples were estimated by measuring their absorbance at 260 and 280 nm and running them in 1% agarose gels.

### Labeling, hybridization, and image quantification

Labeling of cDNA samples, hybridization, spot quantification and data normalization processes were carried out as part of the TIGR Potato Functional Genomics Project using the TIGR Potato 10K cDNA Array, containing ~12,000 potato clones from ESTs that have been re-sequenced and validated (http://jcvi.org/potato/sol_ma_microarrays.shtml). Because of a high sequence similarity among the *Solanaceae* family, potato (*Solanum tuberosum*) probes can be successfully used to detect transcripts of other *Solanaceae* (such as tobacco) plants on microarrays (Senthil et al., 2005; Rensink et al., 2005b; Dardick, 2007; Hall et al., 2007).

The details of the protocols are found in Supplementary Data Sheet 1.

### Data analysis

All raw expression data are available at Gene Expression Omnibus (GEO) (Identifier: GSE10482). Using normalized data significantly activated or repressed genes were selected by Rank Products analyses (Breitling et al., 2004). The number of random permutations used for estimation of false discovery rates (FDR) was 5. Genes that were above the selection limit (5% FDR) were chosen as transcriptionally altered ones (Table S1).

For gene enrichment analysis potato EST sequences were downloaded from the EST database of NCBI (http://www.ncbi.nlm.nih.gov/nucest/). With these sequences BLAST X search was performed with a default setting to find *Arabidopsis* homologs in TAIR10 Proteins (https://www.arabidopsis.org/Blast/index.jsp). The best homologs (Table S2) were used for gene enrichment analysis carried out by using agriGO Singular Enrichment Analysis tool (http://bioinfo.cau.edu.cn/agriGO/analysis.php) with default setting (Statistical test method: Fisher, Multi-test adjustment method: Yekutiely, Significance Level: 0.05, Minimum number of mapping entries: 5). From the significantly enriched Gene Ontology (GO) terms those were selected for presentation that were ranked lower in the GO hierarchy, referring to more specific processes, functions or localization.

For MapMan analysis we used the MapMan tool (Usadel et al., 2005) together with the StuTIGR potato mapping file created by Rotter et al. (2007).

Cluster analyses (appearing in **Figure 2D**) were performed by a Cluster 3.0 software (http://bonsai.hgc.jp/~mdehoon/software/cluster/software.htm#ctv) using k-Means analysis (we chose default settings with 8 gene clusters and 4 treatment clusters).

### Primer design and quantitative RT-PCR analysis of gene expression

For validation of microarray experiments potato EST sequences were downloaded from the NCBI EST database (http://www.ncbi.nlm.nih.gov/nucest/). With these sequences BLASTN searches were performed (http://blast.ncbi.nlm.nih.gov/Blast.cgi) using default settings to find *N. tabacum* homologs of potato EST probes spotted onto the slides. For most similar homologs primers were designed to perform quantitative RT-PCR experiments. Primer sequences for real-time PCR amplifications are shown in Table S3.

Total RNA (2.5 μg) was used for synthesis of 20 μl cDNA. Two and a half μl from a 10-fold dilution of DNase treated cDNA stock was used in each 15 μl reaction using SensiFAST SYBR No-ROX real-time PCR mix (Bioline, UK). Primer concentrations were 3 μM. Real-time PCR amplifications were performed in a DNA Engine Opticon 2 thermocycler (MJ Research, USA). The cycling parameters were: 95°C for 2 min, followed by 40 cycles of 95°C for 15 s, 60°C for 30 s. Measured C(T) values were normalized to actin (GeneBank X69885) as template for internal control values. Sample values were compared to the water injected controls.

## Results and discussion

### Experimental design of bacterial treatments and selection of transcriptionally altered genes

Microarray analyses were performed to investigate transcriptional responses of tobacco plants during bacterial-induced defense reactions, especially during PTI. Inoculum concentrations were consistently adjusted to 10^8^ bacterial cells per milliliter. These suspensions of bacterial cells were injected into middle-aged fully developed tobacco leaves. At this concentration incompatible *P. syringae* wild type bacteria induced HR after about 12–14 h. Injection of compatible *P. tabaci* into the leaves led to tissue softening about 1 day after the inoculation and 3–4 days later triggered late normosensitive necroses (Klement, 1982). In contrast, the *P. syringae hrcC* T3SS deficient mutant and the inactivated *P. tabaci* did not trigger any visible symptoms during the course of the experiments. Water infiltrated samples were used as control for bacterial treatments. Leaf materials were collected at 6 h after inoculations (6 hpi). This sampling time was chosen on the basis of our previous work since PTI develops in tobacco by this time at 20°C as detected by using various marker reactions (Burgyán and Klement, 1979; Ott et al., 1997, 2006; Bozsó et al., 1999, 2005; Klement et al., 1999, 2003; Szatmári et al., 2006, 2014).

In addition, in the case of *P. syringae hrcC*, samples were also collected at 48 hpi to investigate transcriptional changes at a later period of PTI.

### Number of the transcriptionally modified genes and comparison of their changes following various bacterial treatments

The Potato 10K cDNA Array that we used for detecting transcriptional changes contained ~12,000 potato cDNA clones. High sequence similarity within species belonging to the *Solanaceae* family, allowed us using potato probes to detect tobacco transcripts (Senthil et al., 2005; Rensink et al., 2005b; Dardick, 2007; Hall et al., 2007). The cDNA library elements on the microarray were selected from different plant tissues and pathogen (incompatible and compatible) inoculated samples. *N. tabacum* is an allotetraploid plant that contains 48 chromosomes (2*n* = 4*x* = 48) that evolved through interspecific hybridization of the ancestors *Nicotiana sylvestris* (2*n* = 24) and *Nicotiana tomentosiformis* (2*n* = 24) producing homeologous genes. Duplicated genes generated by polyploidization events are referred to as homeologs. A recent study predicts about 81,000–93,000 gene models in *N. tabacum* (Sierro et al., 2014). Importantly, allotetraploidization was thought to take place relatively recently (about 200,000 years ago) and analysis of RNA-seq based transcriptomic data showed that neither the sequences nor the expression levels and functions of the homeologs have diverged remarkably in *N. tabacum* (Bombarely et al., 2012). This means that one cDNA probe can detect transcripts from both homeologous genes, which are transcribed typically in similar fashion. All in all, in spite of the fact that the exact coverage of the used 10K cDNA Array on the tobacco whole transcript cannot be precisely determined the wide source of cDNA probes on the array enabled us detecting representative transcriptomic patterns after different treatments. In the future nearly full coverage transcriptomic data could be obtained by using other platforms (e.g., RNA-seq).

From the normalized transcriptional data we selected transcriptionally modified genes that showed alterations at a maximum of 5% FDR level using the RANK products method (Breitling et al., 2004). Using this selection threshold the lowest significant average fold-change was 2.2X for up-regulation and 0.53X for down-regulation. The numbers of significantly altered genes are shown in Table 1. At 6 hpi numbers of transcriptionally modified genes (activated and repressed genes together) were nearly the same after various treatments. In addition, numbers of up-regulated genes in comparison with numbers of down-regulated genes in particular treatments were also approximately equal. Expression changes were confirmed by quantitative real-time PCR for selected up-or down-regulated genes at 6 hpi in plants developing PTI (Table S3).

**Table 1:** **Number of significantly activated or repressed genes after different bacterial treatments**.

**Treatments**	**6 hpi**		**48 hpi**	
	**Activated^a^**	**Repressed^a^**	**Activated**	**Repressed**
*P. syringae hrcC*	278	269	87	101
*P. syringae*	288	295	Nd^b^^c^	Nd
*P. tab*	297	296	Nd^c^	Nd
*P. tab* inactivated	230	263	Nd	Nd

a Criteria for gene selection are described in the text. Gene activity changes were compared to water-infiltrated samples;

b By 48 hpi P. syringae-infiltrated tissues were collapsed;

c *Not determined*.

**Figure 1:**
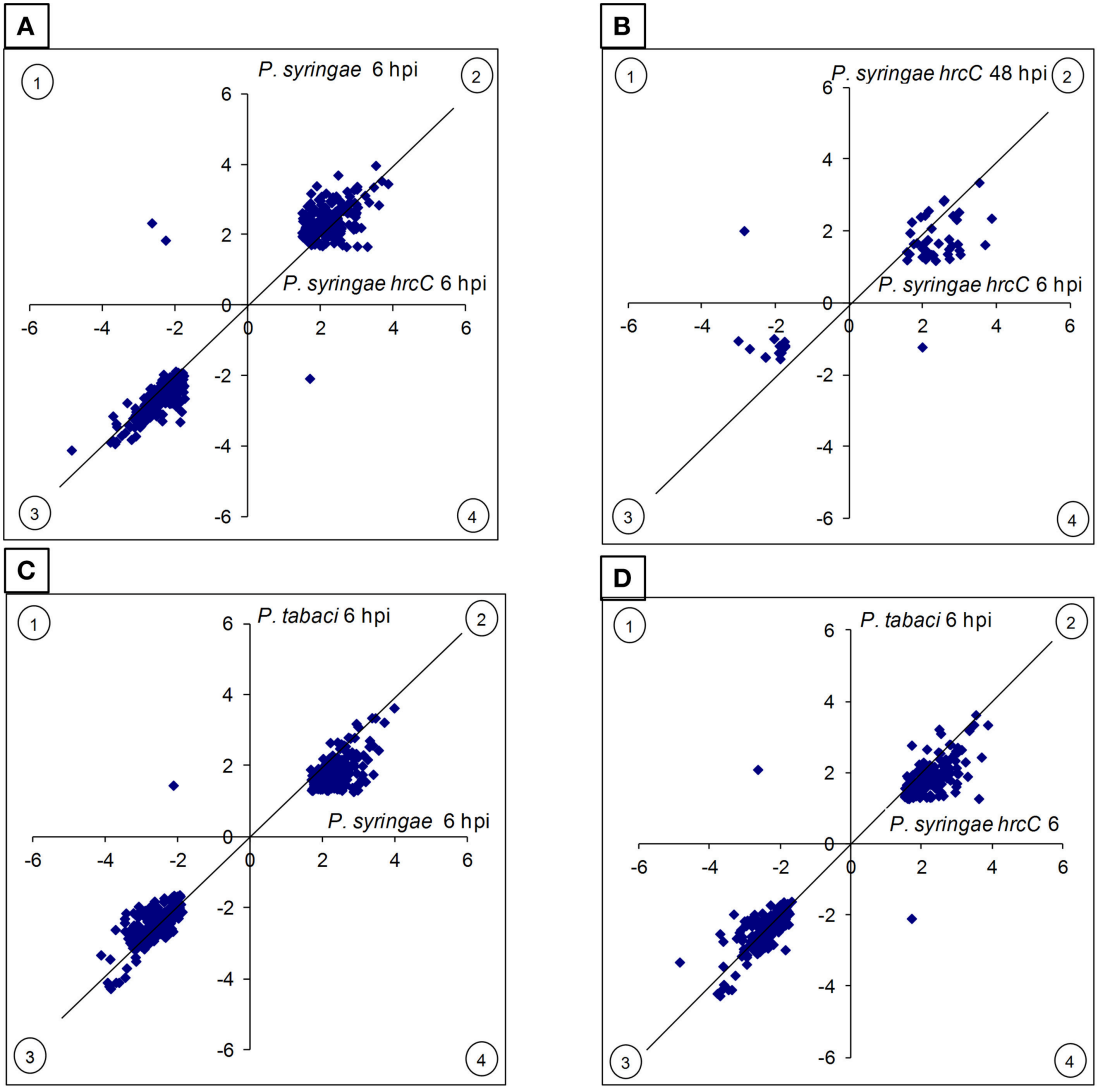
**Comparison of the intensity and directions of gene expression changes induced by different treatments**. The X- and Y axes show average log_2_ transcription activation or repression of those genes of the given treatments that both are differentially expressed when compared to the water-injected control. **(A)** *P. syringae hrcC* (PTI) at 6 h post-inoculation (hpi) vs. *P. syringae* (ETI) at 6 hpi. **(B)** *P. syringae hrcC* (PTI) at 6 hpi vs. *P. syringae hrcC* (PTI) at 48 hpi. **(C)** *P. syringae* (ETI) at 6 hpi vs. *P. tabaci* (compatible) at 6 hpi. **(D)** *P. syringae hrcC* (PTI) at 6 hpi vs. *P. tabaci* (compatible) at 6 hpi. Points in quadrants 2 and 3 shows those genes activated and repressed in the same direction by both treatments, respectively. Points in quadrants 1 and 4 shows those genes that were activated and repressed in the opposite direction in the two treatments. Figure shows results of the average of triplicates.

At 6 hpi the greatest changes in gene activation occurred in ETI-inducing *P. syringae*-injected samples. This was suggested by distribution of fold-change values for overlapping differentially expressed genes (Figure 1) and by the log_2_ average values for up-regulated genes (calculated from the values of all significantly activated genes of a particular treatment, in log_2_ form): *P. syringae* (2.37) > *P. syringae hrcC* (2.16) > *P. tabaci* (1.82). The order of average fold-changes was the same in case of the repressed genes as well: *P. syringae* (−2.47) > *P. syringae hrcC* (−2.25) > *P. tabaci* (−2.18).

**Figure 2:**
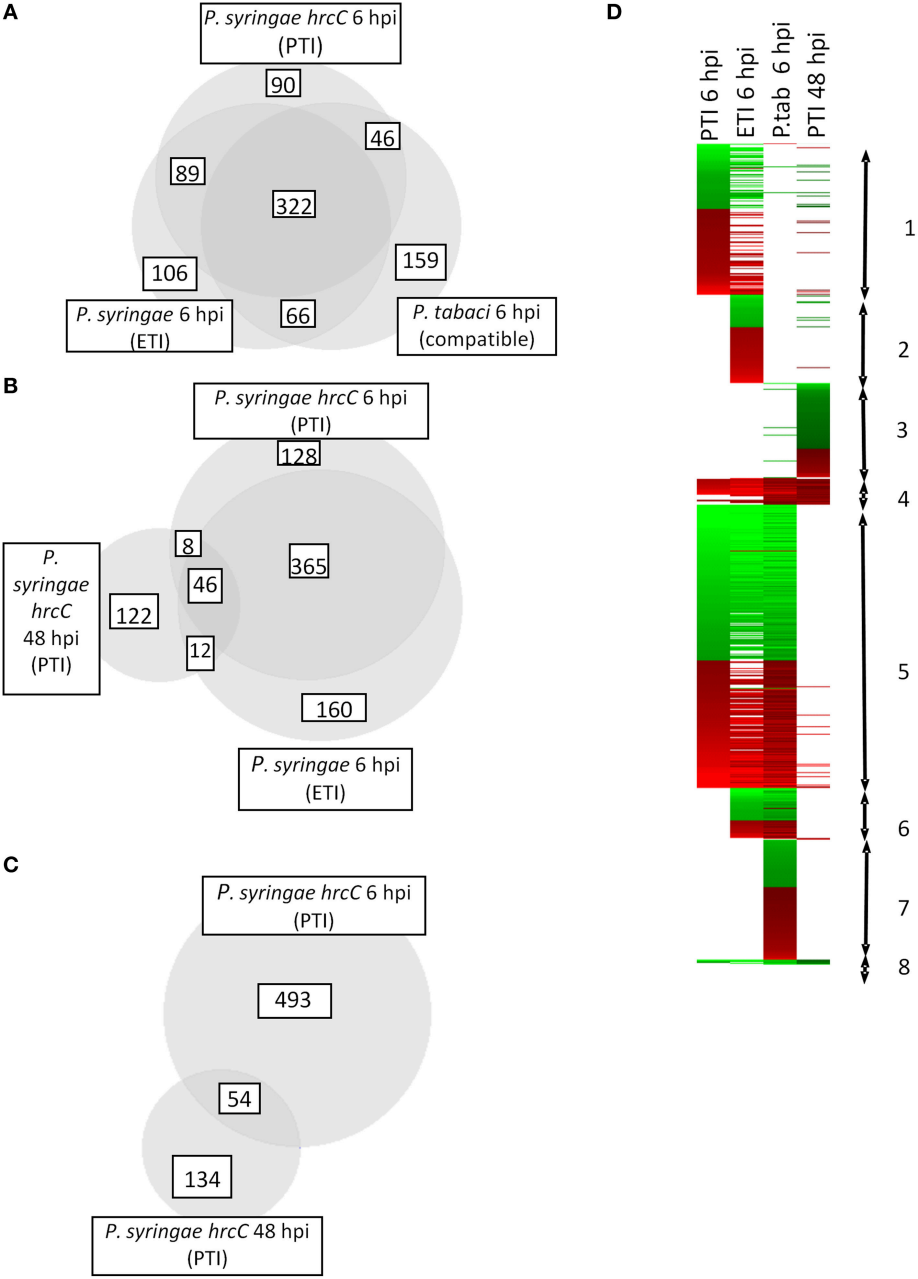
**Number and direction of expression changes induced by different bacterial treatments**. **(A–C)** Number of common and specific genes significantly up- or down-regulated after inoculations. Area-proportional Venn diagrams were produced with help of BioVenn (Hulsen et al., 2008) **(D)** Cluster analysis of transcriptomic alterations induced by different bacterial treatments. Genes appearing in cluster **1** are affected mostly by PTI and ETI at 6 hpi. Some of the genes here are up- or down regulated exclusively during PTI at 6 hpi. Cluster **2** contains genes that are specific mainly to ETI at 6 hpi. Group **3** includes specific genes of late (48 hpi) PTI. Most of the genes belonging to cluster **4** were activated in all samples irrespective of the type of bacterial treatment. Cluster **5** represents genes that changed their transcription at 6 hpi regardless of the type of the treatment. Group **6** contains activated or repressed genes induced by living pathogens with functional Type III secretion system (either ETI inducible *P. syringae* or compatible *P. tabaci*). In cluster **7** there are genes whose transcription was specifically modulated by compatible disease causing *P. tabaci*. Genes belonging to cluster **8** were repressed in all samples irrespective of the type of bacterial treatment. Red and green colors represent up- or down regulation of genes compared to water infiltrated control, respectively.

Comparison of early and late PTI responses to *P. syringae hrcC* (6 vs. 48 hpi) showed that the number of transcriptionally altered genes decreased sharply by 48 hpi (Table 1, Figure 2C). In addition, the intensity of transcriptional changes also got reduced by 48 hpi (Figure 1B). Moreover, the lowest similarity among treatments was observed between the earlier (6 hpi) and later (48 hpi) stages of PTI since only 9.8% (54 of 547) and 29% (54 of 188 of the genes were common, respectively (Figure 2C). These data indicate that at later stages of PTI remarkable reprogramming is occurring compared to the earlier time point.

### Overlapping transcriptomic responses during PTI and ETI

Transcriptomic responses significantly overlap at 6 hpi irrespective of the type of bacterial treatment used (Figures 2A,D). There was a core set of genes that are up- or down regulated after all bacterial inoculations and these genes represent the highest portions of the changes.

The elicitors involved and the outcome of PTI and ETI are apparently different, while PTI is a symptomless response, ETI is accompanied with cell death. In spite of these differences, the highest overlap of common genes was found between PTI and ETI at 6 hpi, since 70–75% of the affected genes were common (411 overlapping genes out of 547 PTI and 583 ETI transcriptionally modified genes).

Generally, the pattern of changes in the transcription of tobacco cells at 6 hpi during PTI and ETI were similar to results obtained with other model plants such as *Arabidopsis* or *Medicago* (Thilmony et al., 2006; Truman et al., 2006; Bozsó et al., 2009).

A typical response during both PTI and ETI at 6 hpi was the activation of phenylpropanoid/phenolics synthesis genes, especially those of lignin biosynthesis, which is characteristic of PTI-associated cell wall strengthening responses (Table S4). The accumulation of phenylpropanoid compounds is a common plant response after bacterial infection (Bestwick et al., 1995; Szatmári et al., 2014). Interestingly, the activity of some of these phenylpropanoid biosynthesis genes are retained even at a later phase of PTI (48 hpi), suggesting the importance of sustained lignin synthesis during PTI.

Intensive expression changes of redox state-related genes including antioxidant/detoxification-associated factors were observable after all types of bacterial inoculation at 6 hpi (Table S5). Several genes involved in ascorbic acid metabolism, which is one of the most important antioxidants in plant cells, were changed in both PTI and ETI. Activation of ascorbate oxidase and decreased expression of GDP-D-mannose 3′,5′-epimerase genes (encoding an enzyme involved in ascorbic acid biosynthesis) suggest lowered ascorbic acid levels at this stage of bacterial infections. Transcription of other antioxidants such as catalase were also declined in ETI and PTI, which implies that some parts of the antioxidant system were suppressed during defense responses. On the other hand, other antioxidants such as non-symbiotic hemoglobin were activated after each bacterial treatment. Peroxidases and glutathione S-transferases involved in antioxidant defense and detoxification of oxidized host molecules were also activated in both ETI and PTI (data not shown).

Another example of common expression changes between PTI and ETI was the general down-regulation of photosynthesis/chloroplast-related genes. Almost all these types of genes were down-regulated at 6 hpi after bacterial treatments during PTI and ETI (Supplementary Data sheet 2). This general blockage in the transcription of photosynthesis-related genes disappeared by 48 hpi in PTI, which suggests the recovery of plant tissues by this time. It seems that the global down-regulation of photosynthesis-related genes is typical of biotic stresses and possibly helps plants to invest resources in immediate defense (Bilgin et al., 2010).

There are twelve ETI-specific genes (transcriptionally not altered at 6 hpi in PTI) that were transcriptionally modified also at a later stage (48 hpi) of PTI (Figure 2B). From these twelve genes seven were down-regulated both in ETI and late PTI. Four of them (STMDF54, STMED79, STMED93, STMGC01) show similarity to recently identified sugar transporters (SWEET genes). Some of these SWEET orthologs were associated with pathogenesis (Chen et al., 2010). A mutation in the promoter region or RNA interference of OsSWEET11 gene confer resistance to *Xanthomonas oryzae* pv. *oryzea* in rice (Chu et al., 2006; Yang et al., 2006). Moreover, a bacterial effector pthXo1 directly interacts with the promoter of OsSWEET11 and activates its transcription leading to an altered sugar transport in the cells (Chen et al., 2010). These data imply that activation of sugar transporters and the sugar efflux should be important in pathogen feeding and that bacteria may modulate sugar transport of their host for their own benefit. Thus, in our experiments the down-regulation of these SWEET homologs in tobacco may contribute to the resistance response. In addition, sugars have been proved to be not only simple nutrients but also signals for defense responses (Bolouri Moghaddam and Van den Ende, 2012). It is known that an increase in the apoplastic hexose-to-sucrose ratio accompanies plant defense responses. Therefore, repression of these genes during ETI and PTI can modulate this signaling process.

### PTI and ETI-specific transcriptomic changes

#### Genes regulated during PTI and ETI in opposite directions at 6 hpi

Besides overlapping genes at 6 hpi, there were some special sets of genes that are transcriptionally modulated by only one type of interaction. Interestingly, within the group of overlapping genes between these two treatments there were only three that were regulated in opposite directions (activated in one but repressed in the other defense reaction compared to water infiltrated control, Figure 1A). One of these genes (STMCZ10; TC207121) has a carboxylic esterase-lipase domain (cl21494) and activated 3.3X (1.71 in log_2_ form) in PTI but repressed during ETI 0.24X (-2.08). Carboxylesterases hydrolyze esters of short-chain fatty acids and have roles in animals ranging from signal transduction to xenobiotic detoxification (Marshall et al., 2003). A BLASTX search showed that this gene is homologous to 2-hydroxyisoflavanone dehydratases that are involved in leguminous isoflavone biosynthesis (Akashi et al., 2005). However, in spite of their activation during defense reactions (Kaschani et al., 2009) the role of this gene product in a non-leguminous plant is not known. The second gene (STMCU44) that was regulated in opposite directions is a homolog of *Arabidopsis* PGR5-like protein 1A (AT4G22890) that is repressed at 6 hpi in PTI (0.16X, −2.64 in log_2_ form) and activated in ETI (5X, 2.34 in log_2_ form). This gene is a transmembrane protein present in thylakoids of chloroplasts. PGRL1 and PGR5 interact physically and associate with photosystem I (PSI). PGRL proteins participate in regulating the electron flow during photosynthesis. It is worth to note that in *P. tabaci*-treated tissues the regulation of both of these above mentioned genes were similar to ETI induced by living *P. syringae*, which suggests that the expression of these genes may depend on the presence of an intact T3SS. The third gene (STMCY19) that was regulated in opposite directions in PTI and ETI has no known function and was down-regulated in PTI (0.21X, −2.26 in log_2_ form) but up-regulated in ETI (3.6X, 1.85 in log_2_ form). These three counter regulated genes may contribute to specific outcomes of PTI and ETI and possible roles of these genes in plant defense will require further studies.

#### Gene enrichment analysis of PTI and ETI specific genes

Several genes investigated at 6 hpi were differentially-regulated exclusively in PTI or in ETI (Figures 2A,D). In these cases transcription of a particular gene was either up- or down-regulated in samples belonging to one treatment but exhibited no change in samples belonging to the other. These treatment-specific genes may confer specificity to the particular defense reaction. At 6 hpi 136 (84 activated and 52 repressed) genes showed altered expression in PTI but not in ETI and 172 (93 activated 79 repressed) were transcriptionally modified in ETI but not during PTI (Table S6). Enrichment analyses were performed with genes up or down-regulated specifically in PTI or ETI to find if there are any specific affected pathways at 6 hpi. Interestingly, the results of Singular Enrichment Analysis of AGRIGO website suggested abiotic stress-related genes to be predominantly altered during both PTI and ETI (Tables 2, 3). While the ETI specific changes have similarities to wound or cold responses, in PTI the salt, heat, osmotic stress and cadmium treatment-related genes were abundant. These results support the overlap between a bacteria-induced biotic stress and some abiotic stresses at least at a transcriptomic level (e.g., Narsai et al., 2013).

**Table 2:** **Gene enrichment results of up-regulated ETI specific genes at 6 hpi (*P. syringae* 61 infiltrated)**.

**GO_acc**	**Term**	**Number of the genes^a^**	**FDR^b^**
**BIOLOGICAL PROCESSES (90 GENES)**			
GO:0009611	Response to wounding	11	1.80E-05
GO:0010200	Response to chitin^c^	11	5.00E-05
GO:0002679	Respiratory burst during defense response	6	0.00028
GO:0050832	Defense response to fungus	8	0.001
GO:0009693	Ethylene biosynthetic process	5	0.0034
GO:0034976	Response to endoplasmic reticulum stress	7	0.0058
GO:0043069	Negative regulation of programmed cell death	5	0.0084
GO:0009867	Jasmonic acid mediated signaling pathway	6	0.0099
GO:0009414	Response to water deprivation	7	0.012
GO:0000165	MAPKKK cascade	5	0.016
GO:0009409	Response to cold	8	0.022
GO:0010363	Regulation of plant-type hypersensitive response	6	0.027
GO:0009862	Systemic acquired resistance, salicylic acid mediated signaling pathway	5	0.028
GO:0009738	Abscisic acid mediated signaling pathway	5	0.028
GO:0006612	Protein targeting to membrane	6	0.031
GO:0006633	Fatty acid biosynthetic process	5	0.046
**CELLULAR COMPONENT^d^ (90 GENES)**			
GO:0005886	Plasma membrane	30	0.00012
GO:0055044	Symplast	9	0.028
GO:0009506	Plasmodesma	9	0.028
GO:0005794	Golgi apparatus	9	0.043
GO:0005774	Vacuolar membrane	6	0.049

a *Number of genes associated with the GO term*.

b *FDR<0.05 were selected as significant enrichment*.

c *GO terms highlighted with gray background were specific to ETI at 6 hpi*.

d *Possible localization of the gene product(s)*.

**Table 3:** **Gene enrichment results of up-regulated PTI specific genes at 6 hpi (*P. syringae* 61 *hrcC* infiltrated)**.

**GO_acc**	**Term**	**Number of genes^a^**	**FDR^b^**
**BIOLOGICAL PROCESSES (69 GENES)**			
GO:0009651	Response to salt stress^c^	14	1.60E-06
GO:0046686	Response to cadmium ion	11	2.60E-06
GO:0034976	Response to endoplasmic reticulum stress	9	1.30E-05
GO:0009627	Systemic acquired resistance	9	7.60E-05
GO:0042542	Response to hydrogen peroxide	7	0.00018
GO:0006094	Gluconeogenesis	6	0.00018
GO:0006096	Glycolysis	5	0.004
GO:0006972	Hyperosmotic response	5	0.0066
GO:0009414	Response to water deprivation	6	0.0084
GO:0009408	Response to heat	5	0.012
GO:0009611	Response to wounding	5	0.019
GO:0006499	N-terminal protein myristoylation	6	0.021
**CELLULAR COMPONENT^d^ (69 GENES)**			
GO:0005783	Endoplasmic reticulum	13	3.10E-07
GO:0005886	Plasma membrane	26	1.20E-05
GO:0005774	Vacuolar membrane	10	1.20E-05
GO:0055044	Symplast	11	0.00012
GO:0009506	Plasmodesma	11	0.00012
GO:0048046	Apoplast	7	0.00088
GO:0005829	Cytosol	11	0.018

a *Number of genes associated with the GO term*.

b *FDR<0.05 were selected as significant enrichment*.

c *GO terms highlighted with gray background were specific to PTI at 6 hpi*.

d *Possible localization of the gene product(s)*.

It is not surprising that during ETI, which is accompanied by a hypersensitive response (HR), cell death, and HR-associated genes were enriched. It is well-known that the oxidative burst contributes to cell death development during PCD. Respiratory burst associated genes were also over-represented in ETI. The encoded proteins can participate in the regulation of the oxidative burst, rather than directly producing ROS (e.g., WRKY transcription factor, zinc finger transcriptional regulator, ubiquitin-protein ligase, F-box family protein etc., the whole gene list of the enrichment results is in Table S7).

In spite of the finding that during PTI there is no sustained oxidative burst (Baker and Orlandi, 1995), several oxidative stress response genes were found exclusively in PTI. For example, monodehydroascorbate reductases, with a role in the ascorbate-glutathione cycle to remove toxic H_2_O_2_ were represented here. Activation of these genes in PTI may prepare plants to avoid a subsequent pathogen attack and can be the consequence of temporal H_2_O_2_ accumulation immediately after exposure to microbes (Baker and Orlandi, 1995).

Considering the microbial defense regulation processes jasmonic acid, salicylic acid, and abscisic acid-mediated signaling pathways along with ethylene biosynthesis were enriched exclusively in ETI, implying that these mechanisms are more pronounced in ETI than in PTI (Table 2). Salicylic acid is an important signaling component both in local and systemic defense responses (Yan and Dong, 2014). It seems that the production and role of salicylic acid in PTI may be different in various plant species. In *Arabidopsis* PTI-inducing agents such as *P. syringae hrcC* mutant bacteria are able to induce salicylic acid accumulation by 6 hpi (Tsuda et al., 2008). Conversely, in tobacco leaves *hrcC* mutants are incapable to trigger the same response (Huang et al., 2003, 2006). Moreover, in *Arabidopsis* plants disruption of SA signaling components strongly affected MAMP-triggered gene expression and decreased resistance to *hrcC* mutant *P. syringae* (Huang et al., 2003). However, in SA depleted tobacco leaves PTI-related gene expression was not significantly different from the controls and the bacterial cell number of the mutant bacteria was unaffected compared to wild-type tobacco (Mur et al., 2000; Szatmári et al., 2006). Recent findings indicated that there are very complex regulatory networks between pathways that are dependent on jasmonic acid, ethylene, salicylic acid or phytoalexin-deficient 4 sectors. In case of PTI interactions are rather synergistic, whereas in ETI interactions are more compensatory (Tsuda et al., 2009). The results also showed that all these four regulatory pathways participated in resistance development and in *Arabidopsis* PTI the ethylene regulatory sector contributes principally to network robustness as a negative regulator of the jasmonic acid pathway (Kim et al., 2014).

Regarding subcellular localizations, enrichment of vacuolar membrane or plasmodesma-related genes with altered expression was found both in PTI and ETI (Tables 2, 3). However, marked enrichment of apoplast and cytosol related genes was found only during PTI, whereas enrichment of Golgi apparatus-related ones was a characteristic of ETI only, suggesting that these cell compartments may take part in specific host responses.

We have also performed enrichment analysis on down-regulated genes that were specifically repressed in ETI or PTI (Tables 4, 5). These enrichment results showed that both in ETI and PTI most of the suppressed plant metabolic processes were chloroplast-related ones. It is not surprising because at 6 hpi in both ETI and PTI about half of the down-regulated genes were related to chloroplastic pathways (data not shown, Figure S1). However, there were some differences in these chloroplast-related processes between ETI and PTI that may reflect the magnitude of stress exerted by these defense reactions on chloroplasts. For example, down-regulated starch biosynthesis process-related genes were overrepresented specifically in ETI (Table 4). In PTI chloroplast-located pentose-phosphate pathway and mevalonate-independent isoprenoid biosynthetic pathway-related processes were enriched in the group of specifically repressed genes (Table 5). Interestingly, during the pathogenesis of compatible *P. tabaci* (in contrast with PTI) cytosol-localized mevalonate-dependent isoprenoid biosynthetic pathway genes were activated (see below).

**Table 4:** **Gene enrichment results of down-regulated ETI specific genes at 6 hpi (*P. syringae* 61 infiltrated)**.

**GO_acc**	**Term**	**Number of genes^a^**	**FDR^b^**
**BIOLOGICAL PROCESSES (67 GENES)**			
GO:0019684	Photosynthesis, light reaction^c^	7	0.0015
GO:0009637	Response to blue light	5	0.0015
GO:0006833	Water transport	5	0.0025
GO:0007030	Golgi organization	5	0.004
GO:0009651	Response to salt stress	9	0.005
GO:0019252	Starch biosynthetic process	5	0.005
GO:0006364	rRNA processing	6	0.0053
GO:0015674	di-, tri-valent inorganic cation transport	6	0.0053
GO:0044262	Cellular carbohydrate metabolic process	13	0.0077
GO:0009658	Chloroplast organization	5	0.0082
GO:0016071	mRNA metabolic process	6	0.0088
GO:0015672	Monovalent inorganic cation transport	5	0.0088
GO:0051186	Cofactor metabolic process	8	0.011
GO:0009266	Response to temperature stimulus	9	0.011
GO:0030001	Metal ion transport	7	0.011
GO:0010035	Response to inorganic substance	9	0.022
GO:0008654	Phospholipid biosynthetic process	5	0.047
GO:0009639	Response to red or far red light	5	0.047
GO:0044271	Cellular nitrogen compound biosynthetic process	7	0.048
**CELLULAR COMPONENT^d^ (67 GENES)**			
GO:0009535	Chloroplast thylakoid membrane	9	1.60E-06
GO:0009570	Chloroplast stroma	11	2.70E-06
GO:0009941	Chloroplast envelope	8	0.00061
GO:0016021	Integral to membrane	7	0.035

a *Number of genes associated with the GO term*.

b *FDR<0.05 were selected as significant enrichment*.

c *GO terms highlighted with gray background were specific to ETI at 6 hpi*.

d *Possible localization of the gene product(s)*.

**Table 5:** **Gene enrichment results of down-regulated PTI specific genes at 6 hpi (*P. syringae* 61 *hrcC* infiltrated)**.

**GO_acc**	**Term**	**Number of genes^a^**	**FDR^b^**
**BIOLOGICAL PROCESSES (45 GENES)**			
GO:0009773	Photosynthetic electron transport in photosystem I^c^	5	2.90E-06
GO:0006098	Pentose-phosphate shunt	7	2.90E-06
GO:0019344	Cysteine biosynthetic process	6	2.60E-05
GO:0015994	Chlorophyll metabolic process	5	0.00024
GO:0010027	Thylakoid membrane organization	5	0.00026
GO:0055080	Cation homeostasis	5	0.00038
GO:0019288	Isopentenyl diphosphate biosynthetic process, mevalonate-independent pathway	5	0.00046
GO:0030001	Metal ion transport	5	0.02
GO:0010038	Response to metal ion	5	0.026
**CELLULAR COMPONENT^d^ (45 GENES)**			
GO:0009535	Chloroplast thylakoid membrane	11	5.20E-11
GO:0009570	Chloroplast stroma	10	3.20E-07
GO:0043234	Protein complex	8	0.0092
GO:0009941	Chloroplast envelope	5	0.014

a *Number of genes associated with the GO term*.

b *FDR<0.05 were selected as significant enrichment*.

c *GO terms highlighted with gray background were specific to PTI at 6 hpi*.

d *Possible localization of the gene product(s)*.

#### PTI and ETI specific signaling and proteolytic genes

MAPMAN classification of differentially regulated genes also pointed out several PTI and ETI specific signal transduction genes that may fundamentally influence the outcome of responses at 6 hpi. The most intense signal transduction processes—especially activation—occurred in tissues undergoing ETI. Protein kinase and protein kinase domain-containing genes were overrepresented within the group of genes involved in signal transduction (e.g., in ETI 21 out of 34 genes) suggesting the primary roles of phosphorylation and kinases in the regulation of defense responses against bacteria (Table S8). Both PTI and ETI had their own characteristic set of signaling receptor kinases (Table 6). The importance of protein kinases in PTI regulation was also highlighted in tomato. Rosli et al. (2013) identified 622 flagellin induced genes that were repressed by AvrPto and AvrPtoB effectors. Within the group of these 622 genes kinases were highly enriched (92) and many of them possess known functions in defense responses. In our experiments three down- and one up-regulated GTP-binding protein genes were observable exclusively in ETI. Two of the three repressed ones were predicted to encode chloroplast-localized proteins (STMIL12, AT3G12080 and STMJF72, AT5G57960) based on their similarities to *Arabidopsis* homologs and their down-regulation can be connected to a general repression of chloroplast genes during defense. The activated GTP-binding gene (STMIV50) is an extra-large G-protein whose homolog in *Arabidopsis* (AT4G34390) may modulate plant defense, and whose mutation caused enhanced susceptibility to *P. syringae* (Zhu et al., 2009; Maruta et al., 2015).

**Table 6:** **Signaling and protein degradation associated genes up- or down-regulated at 6 hpi specifically in ETI (*P. syringae* 61 infiltrated leaves) or in PTI (*P. syringae* 61 *hrcC* infiltrated leaves)**.

**id^a^**	**Fold-change^b^**	**Similarity, Function**
**SIGNALING**		
**PTI 6 hpi**		
STMHW88	**3.58**	Receptor-like protein kinase
STMJG46	**3.68**	Receptor-like protein kinase
STMIR02	**5.94**	Receptor-like serine/threonine kinase
STMJC66	**2.93**	Protein kinase
STMJA91	**3.46**	Calreticulin precursor
**ETI 6 hpi**		
STMIO40	**4.96**	NtEIG-E80 protein, PAR1, PAR1 protein
STMIR07	**3.53**	Protein kinase
STMGL52	**3.48**	S-receptor kinase
STMEG05	**3.89**	EF-hand, calcium binding motif
STMHY91	**5.70**	EF-hand, calcium binding motif
STMJH49	**3.48**	EF-hand, calcium binding motif
STMGY49	**3.97**	Extra-large G-protein-like
STMIL12	**4.08**	Phytochrome A signal transduction 1
STMJF72	**3.86**	Phytochrome A signal transduction 2
STMIV50	**0.15**	GTP-binding protein-related
STMEF62	**0.25**	GTP-binding protein-related
STMJL27	**0.24**	GTP-binding protein-related
**PROTEIN DEGRADATION**		
**PTI 6 hpi**		
STMHJ24	**2.83**	Autophagy 7 [*Arabidopsis thaliana*]
STMHR44	**2.69**	Arm repeat-containing protein
STMHK80	**0.30**	Cucumisin-like serine protease
STMCX90	**0.26**	Serin carboxypeptidase-like protein
**ETI 6 hpi**		
STMDZ53	**4.11**	Ubiquitin domain
STMFA02	**4.89**	Ubiquitin interaction motif-containing protein
STMGU22	**3.58**	Zinc finger (C3HC4-type RING finger) family protein
STMID28	**3.89**	Zinc finger (C3HC4-type RING finger) family protein
STMGU22	**3.58**	Zinc finger (C3HC4-type RING finger) family protein
STMCE77	**3.81**	F-box family protein
STMCY90	**0.16**	UBX domain-containing protein
STMCJ34	**0.21**	Cysteine protease precursor
STMDG47	**0.19**	Pre-pro-cysteine proteinase precursor
STMEU11	**0.24**	Cathepsin B-like cysteine proteinase
STMJB45	**0.19**	Cathepsin B-like cysteine proteinase
STMDM72	**0.25**	CLP proteinase like protein

*Gene selection was done by MAPMAN classification. Red and green colors represent up- or down-regulated genes, respectively. Gene expressions are in log_2_ transformed form*.

a *EST identifier of NCBI EST database (http://www.ncbi.nlm.nih.gov/nucest/)*.

b *Gene expression in log_2_ transformed form*.

Another difference was the higher number of altered proteolytic genes during ETI in comparison with PTI at 6 hpi (Table 6). Several members of the proteasomal degradation system were up-regulated specifically in ETI. Interestingly, within the group of repressed proteolytic genes during ETI were some down-regulated cysteine proteases (STMCJ34, STMDG47, STMEU11) that show homology to *Arabidopsis* genes (AT5G60360, AT4G16190, AT1G02305) involved in programmed cell death and senescence. These cysteine protease homologs are vacuole-targeted and there are experimental data available proving that they are involved in hypersensitive cell death (McLellan et al., 2009). Down-regulation of these genes at this phase of ETI in our system is not clear, but may be explained by a difference between the regulation of tobacco and *Arabidopsis*. In our system suppression of these genes may delay progression of cell death.

### PTI-associated genes suppressed by compatible *P. tabaci*

**Figure 3:**
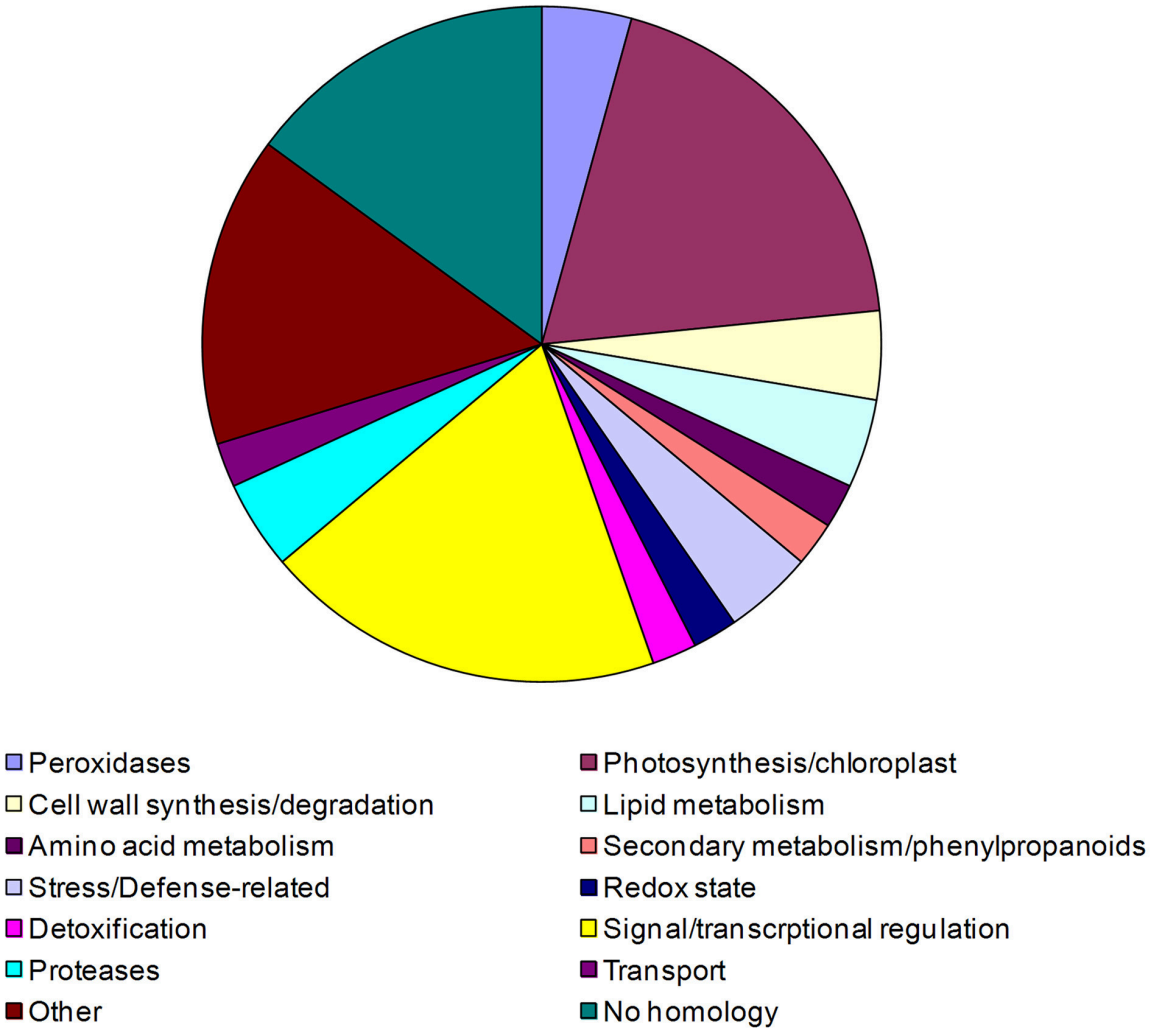
**Pie chart representing percent ratios of putative function-based groups of tobacco genes up or down-regulated during PTI and blocked by compatible *P. tabaci***. 47 PTI activated or repressed tobacco genes were blocked by living *P. tabaci* at 6 hpi. Functional classification of genes was determined by the help of MAPMAN (Rotter et al., 2007). Based on the identified putative functions genes were classified into 14 groups: peroxidases, photosynthesis/chloroplast, cell wall synthesis/degradation, lipid metabolism, amino acid metabolism, secondary metabolism/phenylpropanoids, stress/defense-related, redox state, detoxification, signal/transcriptional regulation, proteases, transport, other no homology. Corresponding percentages are demonstrated in the figure.

Compatible bacteria can influence transcription in the host to inhibit plant defense reactions. To find such genes, we compared transcriptomic changes triggered by living with changes evoked by antibiotic-inactivated bacteria (the latter trigger only PTI). We found only a slight difference between the intensity of the common up- and down-regulated genes at 6 hpi after both bacterial treatments (data not shown). However, living *P. tabaci* actively inhibited the induction or repression of 95 genes in tobacco cells (Table S9). Fourty-seven of these also showed transcriptional changes during PTI in *P. syringae hrcC* treated leaves (Table S9). Therefore, these 47 genes can be considered as a set of PTI genes that are down-regulated during a compatible interaction. Functional classification of these genes (Figure 3) showed that the largest portion is associated with signal transduction and transcriptional regulation processes, e.g., receptor-like kinase, wall-associated kinase, calreticulin (involved in Ca^2+^-regulated signal transduction process), helix-loop-helix-like protein etc. Strikingly, in contrast to all other treatments, no peroxidase gene activation was detectable in living *P. tabaci*-treated tissues. The suppression of the activation of these multifunctional enzymes (peroxidases may function as e.g., pro- and antioxidants, have a role in cell wall modification, etc.) underpins their fundamental roles in defense-related processes (Passardi et al., 2005; Hemetsberger et al., 2012).

As we expected, gene enrichment analysis of genes up-regulated during PTI but blocked by *P. tabaci* also listed several pathogen defense-associated processes (e.g., defense response to fungus, systemic acquired resistance, response to jasmonic acid stimulus, etc., Table 7). The results of analysis imply the importance of the influence on oxidation-reduction by a compatible pathogen. The manipulation of oxidation-reduction exerted by the compatible pathogen may also be important for successful colonization via prevention of a harmful oxidative environment and/or control of redox-related signal processes (e.g., monodehydroascorbate reductases, STMEW20; peroxidase STMFB48; proteins with putative protein-disulfide reductase activity, STMIX36, STMHZ49). *P. tabaci* also blocked genes that are related to the transport of amino acids, nitrate and iron ions, suggesting the significance of these transport processes in resistance responses (Table S7E).

**Table 7:** **Gene enrichment results of up-regulated PTI genes at 6 hpi (*P. syringae* 61 *hrcC* infiltrated) that were blocked by compatible *P. tabaci***.

**GO_acc**	**Term**	**Number of genes^a^**	**FDR^b^**
**BIOLOGICAL PROCESSES (29 GENES)**			
GO:0050832	Defense response to fungus	5	0.00087
GO:0009627	Systemic acquired resistance	5	0.0022
GO:0009753	Response to jasmonic acid stimulus	5	0.0026
GO:0031347	Regulation of defense response	5	0.0039
GO:0023052	Signaling	9	0.0053
GO:0055114	Oxidation reduction	7	0.0053
GO:0006810	Transport	11	0.0058
**CELLULAR COMPONENT^c^ (29 GENES)**			
GO:0005618	Cell wall	5	0.013
GO:0005886	Plasma membrane	11	0.016
GO:0005773	Vacuole	5	0.018
GO:0005576	Extracellular region	9	0.018
GO:0005794	Golgi apparatus	5	0.025

a *Number of genes associated with the GO term*.

b *FDR<0.05 were selected as significant enrichment*.

c *Possible localization of the gene product(s)*.

The enrichment analysis suggested that the periphery of host cells, (e.g., the cell wall and plasma membrane that are involved in contacting, recognition and performing direct defense to attacking pathogens) are the prime targets of a compatible pathogen. In addition, expression of Golgi apparatus and vacuole-associated genes that can be involved in the transport and storage of antimicrobial substances, were also markedly blocked by *P. tabaci*.

Among the down-regulated PTI-related genes whose repression was blocked by *P. tabaci*, photosynthesis/chloroplast-connected genes were mostly represented (Table 8). It is worth to note that an *Arabidopsis* homolog (AT5G58330) of one of these genes, a chloroplast-associated NADP-dependent malate dehydrogenase (STMCK77) has a putative role in chloroplast-originated H_2_O_2_ defense-related signaling. This is accomplished through NADPH-dependent oxidation/reduction processes and peroxisomal catalase activity (Heyno et al., 2014).

**Table 8:** **Gene enrichment results of down-regulated PTI genes at 6 hpi (*P. syringae* 61 *hrcC* infiltrated) that were blocked by compatible *P. tabaci***.

**GO_acc**	**Term**	**Number of genes^a^**	**FDR^b^**
**BIOLOGICAL PROCESSES (16 GENES)**			
GO:0015979	Photosynthesis	5	0.00024
GO:0009416	Response to light stimulus	5	0.0091
GO:0005975	Carbohydrate metabolic process	6	0.014
**CELLULAR COMPONENT^c^ (16 GENES)**			
GO:0009579	Thylakoid	6	1.60E-05
GO:0009941	Chloroplast envelope	6	1.60E-05
GO:0005576	Extracellular region	6	0.026

a *Number of genes associated with the GO term*.

b *FDR<0.05 were selected as significant enrichment*.

c *Possible localization of the gene product(s)*.

### Tobacco genes activated or repressed specifically by compatible *P. tabaci*

Our expression data show that inoculation with compatible *P. tabaci* triggered the most pronounced transcriptional changes at 6 hpi. This treatment resulted in the highest number of unique transcriptional alterations when compared to ETI or PTI (Figure 2A). Besides suppressing transcription of PTI-related host defense genes, the compatible pathogen simultaneously activates some other sets of plant genes. These other sets of activated genes may stimulate pathways in the host cells that make conditions in the apoplast favorable for bacterial multiplication. There were 195 genes that exhibited up- or down regulation in living *P. tabaci*-treated samples but not in samples injected with inactivated *P. tabaci*. From these 195 genes we removed those genes that showed significant alteration also in PTI or ETI at 6 hpi. The remaining 121 genes are considered as factors exclusively affected by living compatible *P. tabaci* at 6 hpi (Table S10).

Among the activated genes abscisic acid-mediated signaling pathway associated genes were overrepresented, appearing in the first three positions of the enrichment analysis results (Table 9). It is known from previous studies that the activation of the abscisic acid pathway by bacterial pathogens enhances susceptibility of plants through suppression of defense-associated pathways (e.g., the salicylic acid pathway; de Torres-Zabala et al., 2007; Mohr and Cahill, 2007; Fan et al., 2009). As in *P. syringae* pv. *tomato*-infected *Arabidopsis* (de Torres-Zabala et al., 2007), genes taking part in regulating the abscisic acid pathway, such as protein phosphatase 2C (STMHS17, AT3G11410), NAC/NAM type transcription factors (STMJD19, AT4G27410; STMEB22, AT1G01720), or leucine zipper motif containing HOMEOBOX 7 (STMHT30, AT2G46680) were activated by *P. tabaci*. These data imply that, manipulation of the abscisic acid pathway by bacterial pathogens can be important also for a *Solanaceae*–*P. syringae* plant–bacterial interaction.

**Table 9:** **Gene enrichment results of up-regulated genes that were specifically activated by compatible *P. tabaci* at 6 hpi (but remained unaffected in PTI, ETI or treatment with inactivated *P. tabaci*)**.

**GO_acc**	**Term**	**Number of genes^a^**	**FDR^b^**
**BIOLOGICAL PROCESSES (66 GENES)**			
GO:0009738	Abscisic acid mediated signaling pathway	8	2.50E-05
GO:0042538	Hyperosmotic salinity response	6	0.00014
GO:0009414	Response to water deprivation	7	0.0018
GO:0019748	Secondary metabolic process	11	0.0037
GO:0006732	Coenzyme metabolic process	7	0.0049
GO:0055086	Nucleobase, nucleoside and nucleotide metabolic process	8	0.0068
GO:0046483	Heterocycle metabolic process	9	0.014
GO:0006066	Alcohol metabolic process	9	0.028
GO:0044255	Cellular lipid metabolic process	10	0.029
GO:0009814	Defense response, incompatible interaction	6	0.032
GO:0048519	Negative regulation of biological process	9	0.044
**CELLULAR COMPONENT^c^ (66 GENES)**			
GO:0005829	Cytosol	15	0.00057
GO:0005737	Cytoplasm	43	0.016

a *Number of genes associated with the GO term*.

b *FDR<0.05 were selected as significant enrichment*.

c *Possible localization of the gene product(s)*.

Specific activation of glutathione peroxidase (GPX) izoenzyme genes were detected in living *P. tabaci*-injected tissues (STMDC64, STMEQ90). It was previously showed that mutation of two chloroplast located *Arabidopsis GPX* genes leads to compromised photooxidative stress tolerance but increased basal resistance to virulent bacteria (Chang et al., 2009). Activation of these genes by *P. tabaci* may decrease the oxidative stress exposure of bacterial cells and contribute to favorable condition for growth.

Three genes of the isoprenoid producing cytosolic mevalonate (MVA) pathway (HMG-CoA reductase: STMEP23, mevalonate kinase: STMEW69, mevalonate diphosphate decarboxylase: STMCB59, STMEG54) were activated specifically by living *P. tabaci* bacteria. (ETI-inducing *P. syringae* activated only a mevalonate kinase (STMCP96) at 6 hpi and none of the genes belonging to this pathway were up-regulated at 6 or 48 hpi during PTI.) This pathway produces isopentenyl diphosphate, via mevalonate as a key intermediate. From isopentenyl diphosphate units different types of isoprenoids such as sesquiterpenes, triterpenes, sterols, brassinosteroids, and ubiquinone are synthesized. Additionally, in plants exists a second, plastid located isoprenoid producing pathway via MEP (2-C-methyl-D-erythritol 4-phosphate) as well. The MEP pathway produces precursors for the biosynthesis of monoterpenes, diterpenes, carotenoids, abscisic acid, strigolactones, gibberellins, and the side chain of chlorophylls and prenylquinones (Rodríguez-Concepción and Boronat, 2015). MEP pathway genes were not influenced by any bacterial treatments in our experimental conditions. To determine if there is any significant impact of the MVA pathway on this plant–bacterial interaction and to find out which metabolites of it could be important would require further studies. One candidate can be brassinosteroids that have already proved to be an inhibitor of PTI (Albrecht et al., 2012).

Cytosol and cytoplasm related genes were enriched among the genes specifically up-regulated by *P. tabaci* (Table 9). This is in contrast with genes blocked by *P. tabaci* whose protein products are mainly associated with cell periphery (Table 7). Thus, living *P. tabaci* on one hand inhibits the expression of cell wall and cell membrane associated genes that are involved in recognition. On the other hand by activating genes that encode cytosol or cytoplasm-localized proteins it is probably able to modify plant processes downstream of recognition. This speaks for a manipulative activity leading to host cell colonization.

Enrichment analysis of genes specifically repressed by *P.tabaci* highlighted that these are mainly associated with defense related processes (Table 10). Interestingly, some of these processes were predominant among genes up-regulated during ETI (but not PTI).

**Table 10:** **Gene enrichment results of down-regulated genes that were specifically repressed by compatible *P. tabaci* at 6 hpi (but remained unaffected in PTI or ETI)**.

**GO_acc**	**Term**	**Number of genes^a^**	**FDR^b^**
**BIOLOGICAL PROCESSES (34 GENES)**			
GO:0000165	MAPKKK cascade^c^	6	3.50E-05
GO:0016117	Carotenoid biosynthetic process	5	3.50E-05
GO:0010114	Response to red light	5	3.50E-05
GO:0015995	Chlorophyll biosynthetic process	5	5.30E-05
GO:0009862	Systemic acquired resistance, salicylic acid mediated signaling pathway	6	5.90E-05
GO:0031348	Negative regulation of defense response	6	7.80E-05
GO:0009867	Jasmonic acid mediated signaling pathway	6	7.80E-05
GO:0010103	Stomatal complex morphogenesis	5	7.80E-05
GO:0006364	rRNA processing	6	0.00013
GO:0010207	Photosystem II assembly	5	0.00013
GO:0010310	Regulation of hydrogen peroxide metabolic process	5	0.00014
GO:0050832	Defense response to fungus	6	0.00014
GO:0010027	Thylakoid membrane organization	5	0.00015
GO:0010363	Regulation of plant-type hypersensitive response	6	0.00019
GO:0032268	Regulation of cellular protein metabolic process	5	0.00021
GO:0006612	Protein targeting to membrane	6	0.00022
GO:0009409	Response to cold	7	0.00022
GO:0019288	Isopentenyl diphosphate biosynthetic process, mevalonate-independent pathway	5	0.00022
GO:0010200	Response to chitin	6	0.00023
GO:0009627	Systemic acquired resistance	6	0.00031
GO:0042742	Defense response to bacterium	5	0.0016
GO:0044275	Cellular carbohydrate catabolic process	5	0.0052
GO:0018193	Peptidyl-amino acid modification	5	0.0079
GO:0009117	Nucleotide metabolic process	5	0.012
GO:0071554	Cell wall organization or biogenesis	5	0.044
**CELLULAR COMPONENT^d^ (34 GENES)**			
GO:0009535	Chloroplast thylakoid membrane	9	3.20E-09
GO:0009570	Chloroplast stroma	8	6.60E-06
GO:0048046	Apoplast	6	9.60E-05
GO:0005618	Cell wall	6	0.00082
GO:0005840	Ribosome	5	0.0024
GO:0009941	Chloroplast envelope	5	0.0042

a *Number of genes associated with the GO term*.

b *FDR<0.05 were selected as significant enrichment*.

c *GO terms highlighted with gray background were terms that were over-represented in ETI at 6 hpi (up-regulated ETI specific genes in Table 2)*.

d *Possible localization of the gene product(s)*.

### Comparison of bacterial-induced transcriptome alterations with abiotic stress-induced gene expression profiles

To see how specific or general the gene expression alterations induced by bacteria are, our data were compared with other published results obtained in response to various abiotic stress agents.

Cold (4°C), heat (35°C), or salt (100 mM NaCl) stress-induced transcriptomic responses (Rensink et al., 2005a) were compared with our gene expression data. As we expected, there was a significant overlap between bacterial and abiotic stress responses. For example, about half of genes altered at 6 hpi during *P. syringae*-triggered PTI or ETI treatments were also modulated by either of the abiotic stress treatments (Table S11A, B). This high overlap suggests that a large portion of plant processes affected during bacterial infections are common to general stress responses, possibly aimed at maintaining cell integrity.

Interestingly, genes specifically activated by living *P. tabaci* showed the broadest overlap with the highly activated abiotic stress-related genes. Some of these genes may be involved in abscisic acid response regulation (Table S11C). This observation further supports the notion that compatible *P. tabaci* activates the abscisic acid response pathway of plant cells to block competing resistance responses.

### Inhibitors of signal transduction pathways affect PTI-related genes

Signaling pathways are key components of defense reactions. Despite the efforts to elucidate signaling processes that are involved in the regulation of genes during the development of PTI, some details of the regulation remain elusive. To investigate the effect of different inhibitors on PTI-related signaling pathways, our microarray experiments were extended using various pharmacological agents. Five different inhibitors were used to block distinct branches of the signaling network: (i) LaCl_2_, a Ca^2+^ channel blocker, (ii) aristolochic acid, a phospholipase A inhibitor, (iii) neomycin, a phospholipase C and D inhibitor, (iv) K252a, a kinase inhibitor, and (v) MG115, a proteasome inhibitor. All of these inhibitors have been successfully used to block plant signaling processes in plants including defense responses (e.g., Adam et al., 1997; Yoon et al., 2000; Lecourieux et al., 2005; Andersson et al., 2006; Seo et al., 2008; Segonzac et al., 2011; Huang et al., 2013; Morimoto et al., 2013).

**Figure 4:**
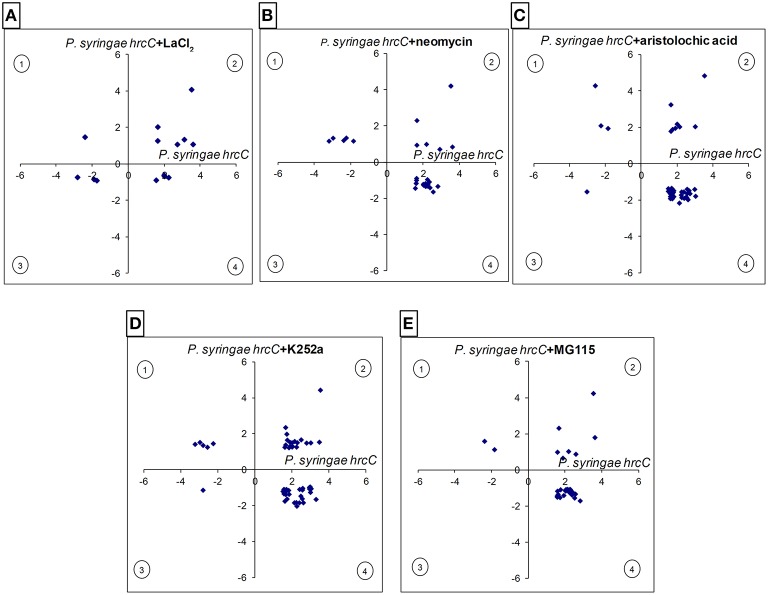
**Comparison of the intensity and directions of gene expression changes caused by various signal inhibitors on PTI-related genes at 6 hpi**. X-axes show average log_2_ transcription activation or repression of PTI-related genes compared to water-injected control (up- or down-regulated in *P. syringe hrcC* infiltrated leaves). Y axes show changes caused by signal inhibitors on PTI-related genes (*P. syringe hrcC*+signal inhibitors) compared to *P. syringe hrcC* (PTI)-injected samples. **(A)** LaCl_2_, Ca^2+^ channel blocker **(B)** neomycin, phospholipase C/D inhibitor **(C)** aristolochic acid, phospholipase A inhibitor. **(D)** K252a, kinase inhibitor **(E)** MG115, proteasome inhibitor. Points in quadrants 2 and 3 show those genes activated and repressed in the same direction in both treatments, respectively. Points in quadrants 1 and 4 show those genes that were activated and repressed in the opposite direction in the two treatments. Figure shows results of the average of triplicates.

**Figure 5:**
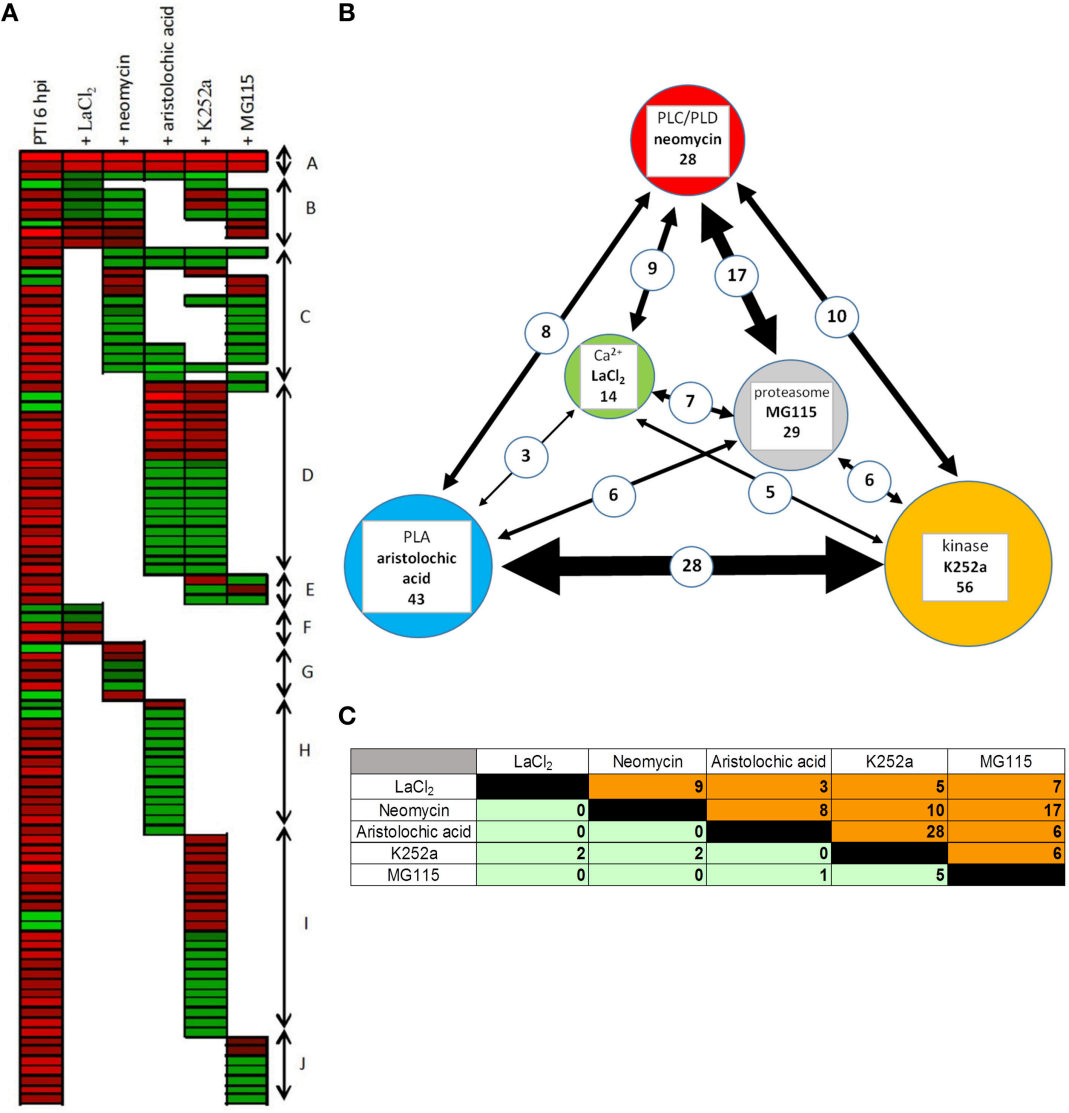
**Effect of different signaling inhibitors on PTI-related gene expression**. **(A)** Expression pattern of PTI-related genes after infiltration together with various signal pathway inhibitors. First column shows transcription of 99 PTI-related genes at 6 hpi in plant leaves after infiltration with *P. syringe hrcC* and compared to water-infiltrated control. Other columns show effects of different signaling pathway inhibitors on the expression of these PTI-related genes. Red and green colors represent up- or down regulation of genes compared to control, respectively. Genes were ranked manually and coloration was carried out by using FiRe 2.2 program. Different letters on the right side of figure mark groups of genes that show similar pattern of expression. (A) Genes whose expression was altered by all inhibitors. (B) PTI-related genes whose expression was not affected by aristolochic acid but influenced by other inhibitors to varying extent. (C) PTI-related genes whose expression was influenced mainly by neomycin and MG115. (D) PTI-related genes whose expression was affected by both aristolochic acid and K252a. (E) PTI-related genes whose expression was influenced by both K252a and MG115. (F–J) Groups represent genes whose expression is influenced by only one inhibitor. **(B)** Interactions between different signal inhibitors in PTI-related gene expressions. Circles represent the used signal inhibitors. Sizes of the circles are proportional to the number of the PTI-related genes influenced by the given inhibitors. Thicknesses of lines between circles are proportional to the number of commonly affected genes, which is indicated with numbers on the line as well. **(C)** Relationships between the effects of different inhibitors on PTI-related gene expressions. Table shows interactions of various signaling pathways during transcriptional regulation of PTI genes. Data presents the number of genes commonly affected by the inhibitors at 6 hpi after infiltration with *P. syringe hrcC*. Orange part of table shows the number of genes that the inhibitors transcriptionally modified to the same direction, while green part of the table shows number of genes modified in opposite directions. Inhibitors were the following: LaCl_2_, Ca^2+^ channel blocker; neomycin, phospholipase C and D inhibitor; aristolochic acid, phospholipase A inhibitor; K252a, kinase inhibitor; MG115, proteasome inhibitor.

The gene expression levels induced by the inhibitor–bacteria (*P. syringae hrcC*) mix were compared to those induced by injecting suspensions of bacteria alone, at 6 hpi. From the resulting differentially expressed genes we selected those that also showed significant alteration in our previous experiments during PTI at 6 hpi. Out of 547 PTI-related genes 99 (18%) were affected significantly at least by one of the signal transduction inhibitors (Table S12). The directions of these changes are shown in Figures 4, 5A. The inhibitors had diverse effects on transcription (i) they could decrease the PTI-induced gene activity changes by reducing the repression or the activation of the transcription (ii) in other cases they could further enhance the PTI-triggered activity changes to the same direction. The obtained patterns suggest that the corresponding signaling pathways may regulate PTI-related genes both positively or negatively. Most frequently the inhibitors repressed transcription of up-regulated PTI-related genes (Figure 5A). The kinase inhibitor had highest impact on gene expression (56 out of 99 genes, 57%), followed by the phospholipase A inhibitor (43 out of 99, 43%), while the Ca^2+^ blocker had the lowest impact on PTI–related transcription (14 out of 99, 14%). (Supplementary Data Sheet 3 contains a simplified model of possible signaling pathways involved in regulation of PTI-related gene expressions).

Cytosolic Ca^2+^ concentration rapidly increases (5–10 min) after recognition of different MAMPs (Lecourieux et al., 2005; Ranf et al., 2011). Prevention of external Ca^2+^ influx by La^3+^ or calcium chelators can inhibit transcription of early PTI inducing genes (Boudsocq et al., 2010; Segonzac et al., 2011). In our experiments LaCl_2_ had only a slight effect on PTI-related gene expression. One possible reason is that 6 hpi is a far too late time point to affect genes whose activity depends on external Ca^2+^ influx.

Previous results revealed that resistance responses in PTI, greatly depend on kinase activity and protein phosphorylation. K252a is a general kinase inhibitor (e.g., it blocks CaM kinase or serine/threonine protein kinase activities) that may potentially inhibit multiple kinases in PTI signaling (Kase et al., 1987; Rüegg and Burgess, 1989; Hashimoto et al., 1991). K252a treatment could block flagellin peptide (flg22)-induced membrane depolarization, and CDPK-regulated gene expression (Boudsocq et al., 2010; Jeworutzki et al., 2010). The principal effect of kinases on PTI was supported by our kinase inhibitor result in concert with a remarkable activation of kinases among signal-related PTI genes (shown in several previous sections).

Three main lipid-hydrolyzing enzyme groups (phospholipase A, C and D) were investigated in our signaling experiments. Aristolochic acid blocks the activity of phospholipase A_2_ (PLA_2_) type enzymes that hydrolyze phospholipids at sn-2 positions and produce free fatty acids (FFAs) and lysophospholipids (Vishwanath et al., 1987). Both PLA_1_ and PLA_2_ type enzymes are able to produce FFAs for oxylipin synthesis (including jasmonic acid) and aristolochic acid was successfully used to block it (Gantet et al., 1998; Engelberth et al., 2001; Ma, 2008). Our transcription results imply that PLA_2_ has a significant role in regulation of PTI, since expression of a relatively high number of genes was modified by the inhibitor. More specific experiments should be conducted to find which PLA_2_ product(s) and target(s) are involved in regulation of plant responses during PTI.

Neomycin is a non-specific phospholipase C (PLC) inhibitor, which is concomitantly able to block phosphatidylcholine-specific phospholipase D (PLD) activity as well (Lipsky and Lietman, 1982; Liscovitch et al., 1991; Andersson et al., 2006). PLC and PLD produce at least two important second messengers (Ca^2+^ release and PA) that can affect the signaling pathway(s) in PTI. The published results suggest that contribution of PLD and PLC to PA formation seems to vary between plant-pathogen systems. PTI elicitors such as the flagellin-derived peptide flg22 enhance PA levels resulting from the activity of PLC (Van der Luit et al., 2000). In other experiments, when bacterial Avr proteins were expressed in *Arabidopsis*, a first wave of PA was produced by PLC and a second one by PLD (Andersson et al., 2006). Our experiments also support that PLC/PLD pathways are involved in PTI gene regulation as neomycin was the third most effective inhibitor of PTI-related genes. This effect might have been accomplished through the modulation of Ca^2+^ and/or PA levels during PTI development.

We used MG115 a potent proteasome inhibitor to block the ubiquitin-mediated degradation of proteins (Vinitsky et al., 1992). As it was mentioned in the introduction, different parts of the PTI-related signaling pathway can be influenced by the proteasomic system. Receptor, transcription factor and transcription initiation complex stability may all depend on proteasome-mediated degradation. Our gene expression results imply that at 6 hpi the proteasomal system is mainly responsible for positive regulation of PTI-related gene expression because most of the genes affected by MG115 inhibitor decreased their transcription (Figure 5A). This could be done directly through the activation of transcription factors (e.g., by degradation of interacting proteins that would normally block the activity of transcription factors) or indirectly by proteolytic cleavage of down-stream signaling elements (such as kinases or phosphatases).

Several genes were affected by more than one inhibitor, suggesting that these signaling elements may belong to the same pathway, and/or alternatively, parallel pathways converge and regulate transcription of the same gene. Highest overlap was found between effects of phospholipase A and kinase inhibitors, affecting transcription of 28 genes out of 99 (28%) PTI-related genes in a similar way (Figures 5B,C). PLA_2_-produced lysophospholipids may promote protein kinase activity (Martiny-Baron and Scherer, 1989; Munnik et al., 1998) that may explain the remarkable overlap between PLA_2_ and kinase inhibitor treatments. PLC/D and proteasome inhibitor treatments also showed remarkably overlapping effects (17 out of 99, 17%). Interactions of these two signaling elements are not clear, but there are some mammalian cell line observations for the regulation of PLC protein levels by proteasomal degradation (Evdonin et al., 2003; Fu et al., 2009). However, it is also feasible that there is no direct interaction between these signaling elements. In these cases downstream signaling processes would be shared which leads to similar transcription patterns.

Genes affected by signal inhibitors are involved in different cellular processes; however, certain inhibitors modulated the activity of specific functional groups of genes typically in one direction (Table S13). Interestingly, kinase inhibitor (K252a) reduced transcription of all detected redox/antioxidant/detoxification-associated genes (protein disulfide isomerase-like genes, catalase, glutathione S-transferase genes), and increased all of the lignin synthesis-associated phenylpropanoid/secondary metabolites genes (phenylalanine ammonia-lyase, N-hydroxycinnamoyl-CoA, tyramine hydroxycinnamoyl transferase, caffeoyl-CoA O-methyltransferase, catechol O-methyltransferase, cinnamyl alcohol dehydrogenase), which suggests that phosphorylation plays positive and negative roles in the transcription of these genes, respectively. In addition, the kinase and phospholipase A inhibitors down-regulated the transcription of all affected signal receptor kinases.

To find possible signaling pathway(s) which can be modified by a compatible pathogen to support its growth, we compared the list of PTI-related genes blocked exclusively by *P. tabaci* and the signal inhibitors. Concerning the 47 *P. tabaci*-blocked PTI-related genes, 11 of them (23%) were also influenced by the signal inhibitors. In nine cases the inhibitors—similar to *P. tabaci*—decreased the transcription of PTI-related genes (Table S14). This data suggests that compatible bacteria may influence the inhibitor-affected pathways to block host gene expression.

## Conclusions

In this study, bacteria such as *P. syringae*, its *hrcC* mutant and *P. tabaci* were infiltrated into tobacco leaves to cause ETI, PTI and susceptible (compatible) interaction, respectively. Host transcriptional changes were assessed at 6 hpi for all treatments and at 48 hpi for PTI. Thus, a susceptible response and two pivotal types of resistance could be compared within one study. It is not surprising that some of our results confirmed previous transcriptomic results since PTI, ETI and compatible responses show similarities in different plant species. Besides calling attention to similarities with other plant-bacterial systems, we also highlighted some details characteristic of tobacco responses. The biological relevance of transcriptomic results manifested in true gene functions need to be confirmed by further biochemical and genetic methods.

Results of our gene expression analyses showed significant resemblance after various bacterial treatments at 6 hpi. At this time point all investigated bacterial treatments altered host transcription in a similar fashion evident in a highly overlapping identity of activated or repressed genes (Table 1, Figures 1, 2). These overlapping transcriptomic changes included diverse plant processes that were directly or indirectly related to plant defense such as cell wall synthesis, proteolysis, signaling (transcription factors, receptor kinases), amino acid synthesis, secondary metabolite synthesis or redox/antioxidant response genes. It could be explained by the fact that PTI inducing PAMPs/MAMPs are released at the beginning of plant-bacterial interactions regardless of the nature of interaction. The transcriptome of ETI in tobacco at 6 hpi stuck out in a sense that gene expression responses were generally more pronounced here, showing notable quantitative differences in comparison with PTI or a susceptible interaction. This observation confirms previous results received by studying other plant–bacterial pathosystems (Tao et al., 2003; Navarro et al., 2004; Bozsó et al., 2009).

Similarities in gene expression between ETI and PTI in tobacco leaves were also indicated by matching activated or repressed transcriptional pathways. It can be exemplified by the activation of phenylpropanoid/lignin synthesis genes and the inhibition of photosynthetic genes. These two detected metabolic responses are known defense-related processes and they have been observed in other plant-pathogen systems previously. Down-regulation of photosynthesis-related genes is typical of stresses and may help host cells to invest resources into immediate defense (Bilgin et al., 2010). Chloroplasts, however, are also needed for the production of defense-related signaling compounds such as salicylic acid, jasmonic acid, secondary metabolites, or reactive oxygen species (Trotta et al., 2014; Delprato et al., 2015). In *Arabidopsis* a chloroplast-localized calcium-sensing receptor (CAS) protein has been identified as a regulator that integrates PTI-related gene activation and repression of chloroplast-associated genes (Nomura et al., 2012). Moreover, crucial roles of chloroplasts in pathogen defense of plants have been supported by the identification of pathogen effectors (including bacterial ones) that specifically target chloroplastic proteins (Bobik and Burch-Smith, 2015). In our experiments there were also several chloroplast/photosynthesis-related genes that were uniquely repressed by only one particular treatment at 6 hpi (Tables 4, 5, 8, 10). These genes may play a role in shaping defense-specific host reactions.

Curiously enough, there were some genes (12) whose transcription changed similarly at a late stage of PTI (48 hpi) and in ETI at 6 hpi. Out of these overlapping genes we emphasize the possible role of sugar transporters (SWEET genes). Four SWEET gene homologs were found to be repressed both in ETI and late PTI. These expression changes suggest that down-regulation of sugar transporter homologs in tobacco may contribute to a resistance response in the host both in ETI and PTI either by reducing the available sugars for bacteria or by modulating sugar dependent signaling processes.

Another similarity between the PTI and ETI transcriptomes was a considerably high number of abiotic stress response-related genes showing altered expression. This was implied by our gene enrichment analyses (Tables 2, 3) and by direct comparisons between our expression results and previously published abiotic transcriptome data (Table S11). It is known from previous results, that the complex interaction between biotic and abiotic stresses is controlled by hormone signaling pathways that orchestrate a network of underlying molecular mechanisms (Atkinson and Urwin, 2012). The considerable overlap of expression changes exhibited by plants after being treated with phytopathogenic bacteria and abiotic stresses may reflect a need in plant cells to maintain cell integrity after various types of stresses. Alternatively, previous abiotic stresses may also leave plant cells in a vulnerable condition, alerting them of potential threats caused by subsequent microbial invasions. Our gene enrichment results also suggested that PTI and ETI may activate distinct sets of transcriptional responses, one resembling gene expression patterns that are characteristic of salt, heat, and osmotic stresses, whereas the other one rather mimicking wound and cold-induced transcriptional responses (Tables 2, 3). Further detailed studies needs to be performed to uncover whether these observation indicate different regulation of PTI and ETI in tobacco at this time point (6 hpi).

Besides broad similarities found in transcriptional regulation between different tobacco–*Pseudomonas* interactions several responses in mRNA expression were identified that were unique to one particular treatment. Three genes were found that were specifically regulated in opposite directions in ETI and PTI at 6 hpi (a carboxylic esterase-lipase domain containing gene, a gene that encodes a photosystem I-associated chloroplast protein and a third gene with unknown functions). So far we could not find any indication of these genes that they would have been connected to plant–microbe interactions but our expression data suggests that they might possess specific roles in a response to microbial pathogens. Enrichment analysis of ETI and PTI specific genes also pointed out some differences between these two types of resistance responses. For example, ETI specific gene expression patterns exhibited the activation of several defense-related genes such as cell death and respiratory burst-connected genes or abscisic acid, jasmonic acid and ethylene pathway-associated genes (Table 2).

A fundamental function of different signaling genes and pathways in resistance development was suggested by the observed expression patterns of signal-related genes and also by our signal inhibitor experiments. Several signaling components have been found in this study that may principally determine the outcome of a host defense response and may differentiate between PTI and ETI. Specific changes in transcription of kinases, receptor-like protein kinases, GTP-binding proteins, and Ca^2+^ signal associated genes were observed at 6 hpi. A crucial role of signaling-related processes was also indicated by the fact that the majority of PTI-connected genes that were suppressed by compatible *P. tabaci* possess signaling functions (Figure 3).

In accord with previous results our data corroborated the pivotal role of phosphorylation and kinases in PTI-associated gene regulation. Since kinases might be involved in multiple steps of signaling pathways such as pathogen sensing (receptor-like kinases), signal transmission (e.g., MAP kinases) and transcription factor regulation, they can be essential components in the control of plant microbial defense mechanisms. Kinases are also typical targets of pathogen effectors (Lindeberg et al., 2012). Genes with protein kinase domain sequences were overrepresented within the group of genes involved in signal transduction during PTI (Table S8). Moreover, pharmacological inhibition of kinases modified the activity of the highest number of PTI-related genes (Figure 5B). Importance of kinases in PTI-associated gene regulation in *Solanaceae* was previously suggested by other authors. For example, transcriptome analysis of tomato demonstrated the overrepresentation of kinases in the group of genes whose expression was modulated by flagellin and blocked by bacterial effectors (Rosli et al., 2013). In *Nicotiana benthamiana* leaves a MAP kinase (NbSIPK) was required for PAMP-induced early gene expression (Segonzac et al., 2011).

Our experiments with signal inhibitors provided a set of particularly informative results since it revealed several regulatory interactions that were not or lesser known before. Besides pointing out important roles of kinases, these experiments also indicated essential functions of phospholipases and proteasomal protein degradation in PTI-related gene expression (Figures 5A,B). Our pharmacological results suggested an involvement of PLA_2_ in PTI-associated gene regulation in tobacco that to our knowledge has not been shown before. Inhibition of PLA_2_ in seagrass greatly reduced the lipopolysaccharide-induced formation of reactive oxygen species (Loucks et al., 2013). Likewise, inhibition of PLA_2_ was able to block reactive oxygen species production and pH changes in ergosterol-treated (fungal PAMP) tobacco cell suspension (Kasparovsky et al., 2004). PLA_2_ is known to produce various second messengers (free fatty acids, lysophospholipids, and oxylipins) thus it can be involved in numerous signaling pathways.

Our signal inhibitor experiments did not cover all signaling elements that may have been involved in PTI-related gene expression. Transcription of about 20% of PTI-regulated genes was affected by the used inhibitors at 6 hpi. Therefore, in addition to those components of signal transduction that were investigated there are some others that can also regulate resistance responses during PTI development. They may control transcription of genes that were not affected by the inhibitors used. These signals might be the following: extracellular alkalinization, membrane potential depolarization and associated ion fluxes (H^+^, Cl^−^, K^+^, and ${\text{NO}}_{3}^{-}$), phosphatases, small GTPases, heterotrimeric G proteins, reactive oxygen- and nitrogen species (Segonzac et al., 2014; Wu et al., 2014).

The signal inhibitor experiments shed light on overlap between different signaling pathways (Figures 5B,C). The notable interactions of PLA_2_ with kinases and of PLC with the proteasomal system are new observations about PTI-related gene regulation. The molecular architecture of these interactions can be explained by known mechanisms (e.g., PLA_2_ deliberates lysophospholipids that may promote protein kinase activity, Martiny-Baron and Scherer, 1989; Munnik et al., 1998) but the exact structure of signal pathway connections is unclear.

Results in this paper including those found in our supplementary data may promote further studies in *Nicotiana* and other solanaceous plants to disclose key mechanisms that shape the development of resistance responses.

## Author contributions

ÁS and ZB designed the experiments. ÁS, ZB, EK, and GB performed the experiments. ÁS, ZB, PO, and MP analyzed the data. ÁS, ZB, PO, and MP wrote the manuscript.

### Conflict of interest statement

The authors declare that the research was conducted in the absence of any commercial or financial relationships that could be construed as a potential conflict of interest.

## References

[B1] Adam A. L. Pike S. Hoyos M. E. Stone J. M. Walker J. C. Novacky A. (1997). Rapid and transient activation of a myelin basic protein kinase in tobacco leaves treated with harpin from *Erwinia amylovora*. Plant Physiol. 115, 853–861. 12223848 10.1104/pp.115.2.853 PMC158546

[B2] Akashi T. Aoki T. Ayabe S. (2005). Molecular and biochemical characterization of 2-hydroxyisoflavanone dehydratase. Involvement of carboxylesterase-like proteins in leguminous isoflavone biosynthesis. Plant Physiol. 137, 882–891. 10.1104/pp.104.056747 15734910 PMC1065389

[B3] Albrecht C. Boutrot F. Segonzac C. Schwessinger B. Gimenez-Ibanez S. Chinchilla D.. (2012). Brassinosteroids inhibit pathogen-associated molecular pattern-triggered immune signaling independent of the receptor kinase BAK1. Proc. Natl. Acad. Sci. U.S.A. 109, 303–308. 10.1073/pnas.1109921108 22087006 PMC3252947

[B4] Andersson M. X. Kourtchenko O. Dangl J. L. Mackey D. Ellerström M. (2006). Phospholipase-dependent signalling during the AvrRpm1- and AvrRpt2-induced disease resistance responses in *Arabidopsis thaliana*. Plant J. 47, 947–959. 10.1111/j.1365-313X.2006.02844.x 16925603

[B5] Anthony R. G. Henriques R. Helfer A. Mészáros T. Rios G. Testerink C.. (2004). A protein kinase target of a PDK1 signalling pathway is involved in root hair growth in *Arabidopsis*. EMBO J. 23, 572–581. 10.1038/sj.emboj.7600068 14749726 PMC1271803

[B6] Asai T. Tena G. Plotnikova J. Willmann M. R. Chiu W. L. Gomez-Gomez L.. (2002). MAP kinase signalling cascade in *Arabidopsis* innate immunity. Nature 415, 977–983. 10.1038/415977a 11875555

[B7] Atkinson N. J. Urwin P. E. (2012). The interaction of plant biotic and abiotic stresses:from genes to the field. J. Exp. Bot. 63, 3523–3543. 10.1093/jxb/ers100 22467407

[B8] Baker C. J. Orlandi E. W. (1995). Active oxygen in plant pathogenesis. Annu. Rev. Phytopathol. 33, 299–321. 10.1146/annurev.py.33.090195.001503 18999963

[B9] Batistič O. Kudla J. (2012). Analysis of calcium signaling pathways in plants. Biochim. Biophys. Acta 1820, 1283–1293. 10.1016/j.bbagen.2011.10.012 22061997

[B10] Bestwick C. S. Bennett M. H. Mansfield J. W. (1995). Hrp mutant of *Pseudomonas syringae* pv. *phaseolicola* induces cell wall alterations but not membrane damage leading to the HR in lettuce (*Lactuca sativa*). Plant Physiol. 108, 503–516. 12228488 10.1104/pp.108.2.503 PMC157369

[B11] Bilgin D. D. Zavala J. A. Zhu J. Clough S. J. Ort D. R. DeLucia E. H. (2010). Biotic stress globally downregulates photosynthesis genes. Plant Cell Environ. 33, 1597–1613. 10.1111/j.1365-3040.2010.02167.x 20444224

[B12] Bobik K. Burch-Smith T. M. (2015). Chloroplast signaling within, between and beyond cells. Front. Plant Sci. 6:781. 10.3389/fpls.2015.00781 26500659 PMC4593955

[B13] Boller T. Felix G. (2009). A renaissance of elicitors: perception of microbe associated molecular patterns and danger signals by pattern-recognition receptors. Annu. Rev. Plant Biol. 60, 379–406. 10.1146/annurev.arplant.57.032905.105346 19400727

[B14] Boller T. He S. Y. (2009). Innate immunity in plants: an arms race between pattern recognition receptors in plants and effectors in microbial pathogens. Science 324, 742–744. 10.1126/science.1171647 19423812 PMC2729760

[B15] Bolouri Moghaddam M. R. Van den Ende W. (2012). Sugars and plant innate immunity. J. Exp. Bot. 63, 3989–3998. 10.1093/jxb/ers129 22553288

[B16] Bombarely A. Edwards K. D. Sanchez-Tamburrino J. Mueller L. A. (2012). Deciphering the complex leaf transcriptome of the allotetraploid species *Nicotiana tabacum*: a phylogenomic perspective. BMC Genomics. 13:406. 10.1186/1471-2164-13-406 22900718 PMC3582432

[B17] Boudsocq M. Sheen J. (2013). CDPKs in immune and stress signaling. Trends Plant Sci. 18, 30–40. 10.1016/j.tplants.2012.08.008 22974587 PMC3534830

[B18] Boudsocq M. Willmann M. R. McCormack M. Lee H. Shan L. He P. (2010). Differential innate immune signalling via Ca^2+^ sensor protein kinases. Nature 464, 418–422. 10.1038/nature08794 20164835 PMC2841715

[B19] Bozsó Z. Maunoury N. Szatmári A. Mergaert P. Ott P. G. Zsíros L. R. Szabó E.. (2009). Transcriptome analysis of bacterially induced basal and hypersensitive response of *Medicago truncatula*. Plant Mol. Biol. 70, 627–646. 10.1007/s11103-009-9496-8 19466566

[B20] Bozsó Z. Ott P. G. Kecskés M. L. Klement Z. (1999). Effect of heat and cycloheximide treatment of tobacco on the ability of *Pseudomonas syringae* pv. *syringae* 61 *hrp/hrmA* mutants to cause HR. Physiol. Mol. Plant Pathol. 55, 215–223. 10.1006/pmpp.1999.0225

[B21] Bozsó Z. Ott P. G. Szatmári A. Czelleng A. Varga G. Besenyei E. (2005). Early detection of bacterium-induced basal resistance in tobacco leaves with diaminobenzidine and dichlorofluorescein diacetate. J. Phytopathol. 153, 596–607. 10.1111/j.1439-0434.2005.01026.x

[B22] Breitling R. Armengaud P. Amtmann A. Herzyk P. (2004). Rank products: a simple, yet powerful, new method to detect differentially regulated genes in replicated microarray experiments. FEBS Lett. 573, 83–92. 10.1016/j.febslet.2004.07.055 15327980

[B23] Burgyán J. Klement Z. (1979). Early induced selective inhibition of incompatible bacteria in tobacco plants. Phytopathol. Mediterr. 18, 153–161.

[B24] Canonne J. Froidure-Nicolas S. Rivas S. (2011). Phospholipases in action during plant defense signaling. Plant Signal. Behav. 6, 13–18. 10.4161/psb.6.1.14037 21248491 PMC3121997

[B25] Chang C. C. Slesak I. Jordá L. Sotnikov A. Melzer M. Miszalski Z.. (2009). *Arabidopsis* chloroplastic glutathione peroxidases play a role in cross talk between photooxidative stress and immune responses. Plant Physiol. 150, 670–683. 10.1104/pp.109.135566 19363092 PMC2689974

[B26] Chen L. Q. Hou B. H. Lalonde S. Takanaga H. Hartung M. L. Qu X. Q.. (2010). Sugar transporters for intercellular exchange and nutrition of pathogens. Nature 468, 527–532. 10.1038/nature09606 21107422 PMC3000469

[B27] Chu Z. Yuan M. Yao J. Ge X. Yuan B. Xu C.. (2006). Promoter mutations of an essential gene for pollen development result in disease resistance in rice. Genes Dev. 20, 1250–1255. 10.1101/gad.1416306 16648463 PMC1472899

[B28] Dardick C. (2007). Comparative expression profiling of *Nicotiana benthamiana* leaves systemically infected with three fruit tree viruses. Mol. Plant Microbe Interact. 20, 1004–1017. 10.1094/MPMI-20-8-1004 17722703

[B29] Delprato M. L. Krapp A. R. Carrillo N. (2015). Green light to plant responses to pathogens: the role of chloroplast light-dependent signaling in biotic stress. Photochem. Photobiol. 91, 1004–1011. 10.1111/php.12466 25989185

[B30] de Torres-Zabala M. Truman W. Bennett M. H. Lafforgue G. Mansfield J. W. Rodriguez Egea P.. (2007). *Pseudomonas syringae* pv. *tomato* hijacks the *Arabidopsis* abscisic acid signalling pathway to cause disease. EMBO J. 26, 1434–1443. 10.1038/sj.emboj.7601575 17304219 PMC1817624

[B31] Dow M. Newman M. A. von Roepenack E. (2000). The induction and modulation of plant defense responses by bacterial lipopolysaccharides. Annu. Rev. Phytopathol. 38, 241–261. 10.1146/annurev.phyto.38.1.241 11701843

[B32] Engelberth J. Koch T. Schüler G. Bachmann N. Rechtenbach J. Boland W. (2001). Ion channel-forming alamethicin is a potent elicitor of volatile biosynthesis and tendril coiling. Cross talk between jasmonate and salicylate signaling in lima bean. Plant Physiol. 125, 369–377. 10.1104/pp.125.1.369 11154344 PMC61017

[B33] Evdonin A. L. Tsupkina N. V. Nikol'skii N. N. Medvedeva N. D. (2003). Ubiquitinylation and proteasome-dependent degradation of the phosphoinositide-specific phospholipase C gamma 1 in A-431 cells. Dokl. Biol. Sci. 392, 456–457. 10.1023/A:1026152527343 14650885

[B34] Fan J. Hill L. Crooks C. Doerner P. Lamb C. (2009). Abscisic acid has a key role in modulating diverse plant-pathogen interactions. Plant Physiol. 150, 1750–1761. 10.1104/pp.109.137943 19571312 PMC2719142

[B35] Felix G. Boller T. (2003). Molecular sensing of bacteria in plants. The highly conserved RNA-binding motif RNP-1 of bacterial cold shock proteins is recognized as an elicitor signal in tobacco. J. Biol. Chem. 278, 6201–6208. 10.1074/jbc.M209880200 12471032

[B36] Fu D. Ma Y. Wu W. Zhu X. Jia C. Zhao Q.. (2009). Cell-cycle-dependent PC-PLC regulation by APC/C(Cdc20)-mediated ubiquitin-proteasome pathway. J. Cell. Biochem. 107, 686–696. 10.1002/jcb.22163 19347873

[B37] Furlan G. Klinkenberg J. Trujillom M. (2012). Regulation of plant immune receptors by ubiquitination. Front. Plant Sci. 3:238. 10.3389/fpls.2012.00238 23109936 PMC3479402

[B38] Gantet P. Imbault N. Thiersault M. Doireau P. (1998). Necessity of a functional octadecanoic pathway for indole alkaloid synthesis by *Catharanthus roseus* cell suspensions cultured in an auxin-starved medium. Plant Cell Physiol. 39, 220–225. 10.1093/oxfordjournals.pcp.a029360

[B39] Gardiner J. Andreeva Z. Barton D. Ritchie A. Overall R. Marc J. (2008). The phospholipase A inhibitor, aristolochic acid, disrupts cortical microtubule arrays and root growth in *Arabidopsis*. Plant Biol. (Stuttg) 10, 725–731. 10.1111/j.1438-8677.2008.00090.x 18950430

[B40] Gassmann W. Bhattacharjee S. (2012). Effector-triggered immunity signaling: from gene-for-gene pathways to protein-protein interaction networks. Mol. Plant Microbe Interact. 25, 862–868. 10.1094/MPMI-01-12-0024-IA 22414439

[B41] Geng F. Wenzel S. Tansey W. P. (2012). Ubiquitin and proteasomes in transcription. Annu. Rev. Biochem. 81, 177–201. 10.1146/annurev-biochem-052110-120012 22404630 PMC3637986

[B42] Groll M. Schellenberg B. Bachmann A. S. Archer C. R. Huber R. Powell T. K.. (2008). A plant pathogen virulence factor inhibits the eukaryotic proteasome by a novel mechanism. Nature 452, 755–758. 10.1038/nature06782 18401409

[B43] Gust A. A. Biswas R. Lenz H. D. Rauhut T. Ranf S. Kemmerling B.. (2007). Bacteria-derived peptidoglycans constitute pathogen-associated molecular patterns triggering innate immunity in *Arabidopsis*. J. Biol. Chem. 282, 32338–32348. 10.1074/jbc.M704886200 17761682

[B44] Hall H. C. Samuel M. A. Ellis B. E. (2007). SIPK conditions transcriptional responses unique to either bacterial or oomycete elicitation in tobacco. Mol. Plant Pathol. 8, 581–594. 10.1111/j.1364-3703.2007.00424.x 20507523

[B45] Hashimoto Y. Nakayama T. Teramoto T. Kato H. Watanabe T. Kinoshita M.. (1991). Potent and preferential inhibition of Ca^2+^/calmodulin-dependent protein kinase II by K252a and its derivative, KT5926. Biochem. Biophys. Res. Commun. 181, 423–429. 10.1016/S0006-291X(05)81436-6 1659814

[B46] Hauck P. Thilmony R. He S. Y. (2003). A *Pseudomonas syringae* type III effector suppresses cell wall-based extracellular defense in susceptible *Arabidopsis* plants. Proc. Natl. Acad. Sci. U.S.A. 100, 8577–8582. 10.1073/pnas.1431173100 12817082 PMC166271

[B47] Hemetsberger C. Herrberger C. Zechmann B. Hillmer M. Doehlemann G. (2012). The *Ustilago maydis* effector Pep1 suppresses plant immunity by inhibition of host peroxidase activity. PLoS Pathog. 8:e1002684. 10.1371/journal.ppat.1002684 22589719 PMC3349748

[B48] Heu S. Hutcheson S. W. (1993). Nucleotide sequence and properties of the *hrmA* locus associated with the *Pseudomonas syringae* pv. *syringae* 61 *hrp* gene cluster. Mol. Plant Microbe Interact. 6, 553–564. 10.1094/MPMI-6-553 8274770

[B49] Heyno E. Innocenti G. Lemaire S. D. Issakidis-Bourguet E. Krieger-Liszkay A. (2014). Putative role of the malate valve enzyme NADP-malate dehydrogenase in H_2_O_2_ signalling in *Arabidopsis*. Philos. Trans. R. Soc. Lond. B Biol. Sci. 369:20130228. 10.1098/rstb.2013.0228 24591715 PMC3949393

[B50] Huang J. Cardoza Y. J. Schmelz E. A. Raina R. Engelberth J. Tumlinson J. H. (2003). Differential volatile emissions and salicylic acid levels from tobacco plants in response to different strains of *Pseudomonas syringae*. Planta 217, 767–775. 10.1007/s00425-003-1039-y 12712338

[B51] Huang W. E. Huang L. Preston G. M. Naylor M. Carr J. P. Li Y.. (2006). Quantitative *in situ* assay of salicylic acid in tobacco leaves using a genetically modified biosensor strain of *Acinetobacter* sp. ADP1. Plant J. 46, 1073–1083. 10.1111/j.1365-313X.2006.02758.x 16805738

[B52] Huang W. Miao M. Kud J. Niu X. Ouyang B. Zhang J.. (2013). SlNAC1, a stress-related transcription factor, is fine-tuned on both the transcriptional and the post-translational level. New Phytol. 197, 1214–1224. 10.1111/nph.12096 23278405

[B53] Hulsen T. de Vlieg J. Alkema W. (2008). BioVenn - a web application for the comparison and visualization of biological lists using area-proportional Venn diagrams. BMC Genomics 9:488 10.1186/1471-2164-9-488 18925949 PMC2584113

[B54] Jakobek J. L. Smith J. A. Lindgren P. B. (1993). Suppression of bean defense responses by *Pseudomonas syringae*. Plant Cell 5, 57–63. 10.1105/tpc.5.1.57 12271016 PMC160250

[B55] Jeworutzki E. Roelfsema M. R. Anschütz U. Krol E. Elzenga J. T. Felix G.. (2010). Early signaling through the *Arabidopsis* pattern recognition receptors FLS2 and EFR involves Ca-associated opening of plasma membrane anion channels. Plant J. 62, 367–378. 10.1111/j.1365-313X.2010.04155.x 20113440

[B56] Jones J. D. G. Dangl J. L. (2006). The plant immune system. Nature 444, 323–329. 10.1038/nature05286 17108957

[B57] Jung J. Kumar K. Lee H. Y. Park Y. I. Cho H. T. Ryu S. B.. (2012). Translocation of phospholipase A_2_α to apoplasts is modulated by developmental stages and bacterial infection in *Arabidopsis*. Front. Plant Sci. 3:126. 10.3389/fpls.2012.00126 22719742 PMC3376726

[B58] Kaschani F. Gu C. Niessen S. Hoover H. Cravatt B. F. van der Hoorn R. A. (2009). Diversity of serine hydrolase activities of unchallenged and botrytis-infected *Arabidopsis thaliana*. Mol. Cell. Proteomics 8, 1082–1093. 10.1074/mcp.M800494-MCP200 19136719 PMC2689769

[B59] Kase H. Iwahashi K. Nakanishi S. Matsuda Y. Yamada K. Takahashi M.. (1987). K-252 compounds, novel and potent inhibitors of protein kinase C and cyclic nucleotide-dependent protein kinases. Biochem. Biophys. Res. Commun. 142, 436–440. 10.1016/0006-291X(87)90293-2 3028414

[B60] Kasparovsky T. Blein J. P. Mikes V. (2004). Ergosterol elicits oxidative burst in tobacco cells via phospholipase A_2_ and protein kinase C signal pathway. Plant Physiol. Biochem. 42, 429–435. 10.1016/j.plaphy.2004.04.003 15191747

[B61] Keshavarzi M. Soylu S. Brown I. Bonas U. Nicole M. Rossiter J.. (2004). Basal defenses induced in pepper by lipopolysaccharides are suppressed by *Xanthomonas campestris* pv. vesicatoria. Mol. Plant Microbe Interact. 17, 805–815. 10.1094/MPMI.2004.17.7.805 15242175

[B62] Kim Y. Tsuda K. Igarashi D. Hillmer R. A. Sakakibara H. Myers C. L.. (2014). Mechanisms underlying robustness and tunability in a plant immune signaling network. Cell Host Microbe 15, 84–94. 10.1016/j.chom.2013.12.002 24439900 PMC4075322

[B63] King E. O. Ward M. K. Raney D. E. (1954). Two simple media for the demonstration of pyocyanine and fluorescein. J. Lab. Clin. Med. 22, 301–307. 13184240

[B64] Klement Z. (1963). Rapid detection of the pathogenicity of phytophatogenic pseudomonads. Nature 199, 299–300. 10.1038/199299b0 14076706

[B65] Klement Z. (1982). Hypersensitivity, in Phytopathogenic Prokaryotes II, eds Mount M. S. Lacy G. H. (New York, NY: Academic Press), 149–177.

[B66] Klement Z. (1990). Generally used pathophysiological methods, in Methods in Phytobacteriology, eds Klement Z. Rudolph K. Sands D. C. (Budapest: Akadémi Kiadó), 96–121.

[B67] Klement Z. Bozsó Z. Kecskés M. L. Besenyei E. Czelleng A. Ott P. G. (2003). Local early induced resistance of plants as the first line of defence against bacteria. Pest Manag. Sci. 59, 465–474. 10.1002/ps.694 12701709

[B68] Klement Z. Bozsó Z. Ott P. G. Kecskés M. L. Rudolph K. (1999). Symptomless resistant response instead of the hypersensitive reaction in tobacco leaves after infiltration of heterologous pathovars of *Pseudomonas syringae*. J. Phytopathol. 147, 467–475. 10.1111/j.1439-0434.1999.tb03852.x

[B69] Kunze G. Zipfel C. Robatzek S. Niehaus K. Boller T. Felix G. (2004). The N terminus of bacterial elongation factor Tu elicits innate immunity in *Arabidopsis* plants. Plant Cell 16, 3496–3507. 10.1105/tpc.104.026765 15548740 PMC535888

[B70] Lecourieux D. Lamotte O. Bourque S. Wendehenne D. Mazars C. Ranjeva R. (2005). Proteinaceous and oligosaccharidic elicitors induce different calcium signatures in the nucleus of tobacco cells. Cell Calcium 8, 527–538. 10.1016/j.ceca.2005.06.036 16198416

[B71] Lindeberg M. Cunnac S. Collmer A. (2012). *Pseudomonas syringae* type III effector repertoires: last words in endless arguments. Trends Microbiol. 20, 199–208. 10.1016/j.tim.2012.01.003 22341410

[B72] Lipsky J. J. Lietman P. S. (1982). Aminoglycoside inhibition of a renal phosphatidylinositol phospholipase C. J. Pharmacol. Exp. Ther. 220, 287–292. 6276533

[B73] Liscovitch M. Chalifa V. Danin M. Eli Y. (1991). Inhibition of neural phospholipase D activity by aminoglycoside antibiotics. Biochem. J. 279, 319–321. 10.1042/bj2790319 1930152 PMC1151584

[B74] Liu H. Stone S. L. (2011). E3 ubiquitin ligases and abscisic acid signaling. Plant Signal. Behav. 6, 344–348. 10.4161/psb.6.3.13914 21364320 PMC3142413

[B75] Loucks K. Waddell D. Ross C. (2013). Lipopolysaccharides elicit an oxidative burst as a component of the innate immune system in the seagrass *Thalassia testudinum*. Plant Physiol. Biochem. 70, 295–303. 10.1016/j.plaphy.2013.05.023 23807482

[B76] Lu D. Lin W. Gao X. Wu S. Cheng C. Avila J.. (2011). Direct ubiquitination of pattern recognition receptor FLS2 attenuates plant innate immunity. Science 332, 1439–1442. 10.1126/science.1204903 21680842 PMC3243913

[B77] Ma C. J. (2008). Cellulase elicitor induced accumulation of capsidiol in *Capsicum annuum*. L. suspension cultures. Biotechnol. Lett. 30, 961–965. 10.1007/s10529-007-9624-y 18066498

[B78] Mackey D. McFall A. J. (2006). MAMPs and MIMPs: proposed classifications for inducers of innate immunity. Mol. Microbiol. 61, 1365–1371. 10.1111/j.1365-2958.2006.05311.x 16899081

[B79] Marino D. Peeters N. Rivas S. (2012). Ubiquitination during plant immune signaling. Plant Physiol. 160, 15–27. 10.1104/pp.112.199281 22689893 PMC3440193

[B80] Marshall S. D. Putterill J. J. Plummer K. M. Newcomb R. D. (2003). The carboxylesterase gene family from *Arabidopsis thaliana*. J. Mol. Evol. 57, 487–500. 10.1007/s00239-003-2492-8 14738307

[B81] Martiny-Baron G. Scherer G. F. (1989). Phospholipid-stimulated protein kinase in plants. J. Biol. Chem. 264, 18052–11859. 2530218

[B82] Maruta N. Trusov Y. Brenyah E. Parekh U. Botella J. R. (2015). Membrane-localized extra-large G-proteins and Gβγ of the heterotrimeric G proteins form functional complexes engaged in plant immunity in *Arabidopsis*. Plant Physiol. 167, 1004–1016. 10.1104/pp.114.255703 25588736 PMC4348786

[B83] McLellan H. Gilroy E. M. Yun B. W. Birch P. R. Loake G. J. (2009). Functional redundancy in the *Arabidopsis* Cathepsin B gene family contributes to basal defence, the hypersensitive response and senescence. New Phytol. 183, 408–418. 10.1111/j.1469-8137.2009.02865.x 19453434

[B84] Mohr P. G. Cahill D. M. (2007). Suppression by ABA of salicylic acid and lignin accumulation and the expression of multiple genes, in *Arabidopsis* infected with *Pseudomonas syringae* pv. tomato. Funct. Integr. Genomics 7, 181–191. 10.1007/s10142-006-0041-4 17149585

[B85] Morimoto K. Mizoi J. Qin F. Kim J. S. Sato H. Osakabe Y. (2013). Stabilization of *Arabidopsis* DREB2A is required but not sufficient for the induction of target genes under conditions of stress. PLoS ONE 8:e80457 10.1371/journal.pone.0080457 24376497 PMC3871162

[B86] Munnik T. Irvine R. F. Musgrave A. (1998). Phospholipid signalling in plants. Biochim. Biophys. Acta 1389, 222–272. 10.1016/S0005-2760(97)00158-6 9512651

[B87] Munnik T. Testerink C. (2009). Plant phospholipid signaling: “in a nutshell”. J. Lipid Res. 50(Suppl.), S260–S265. 10.1194/jlr.R800098-JLR200 19098305 PMC2674723

[B88] Mur L. A. Brown I. R. Darby R. M. Bestwick C. S. Bi Y. M. Mansfield J. W.. (2000). A loss of resistance to avirulent bacterial pathogens in tobacco is associated with the attenuation of a salicylic acid-potentiated oxidative burst. Plant J. 23, 609–621. 10.1046/j.1365-313x.2000.00825.x 10972887

[B89] Narsai R. Wang C. Chen J. Wu J. Shou H. Whelan J. (2013). Antagonistic, overlapping and distinct responses to biotic stress in rice (*Oryza sativa*) and interactions with abiotic stress. BMC Genomics. 14:93. 10.1186/1471-2164-14-93 23398910 PMC3616870

[B90] Navarro L. Zipfel C. Rowland O. Keller I. Robatzek S. Boller T.. (2004). The transcriptional innate immune response to flg22. Interplay and overlap with Avr gene-dependent defense responses and bacterial pathogenesis. Plant Physiol. 135, 1113–1128. 10.1104/pp.103.036749 15181213 PMC514144

[B91] Newman M. A. Sundelin T. Nielsen J. T. Erbs G. (2013). MAMP (microbe-associated molecular pattern) triggered immunity in plants. Front. Plant Sci. 4:139. 10.3389/fpls.2013.00139 23720666 PMC3655273

[B92] Nicaise V. Roux M. Zipfel C. (2009). Recent advances in PAMP-triggered immunity against bacteria: pattern recognition receptors watch over and raise the alarm. Plant Physiol. 150, 1638–1647. 10.1104/pp.109.139709 19561123 PMC2719144

[B93] Nomura H. Komori T. Uemura S. Kanda Y. Shimotani K. Nakai K.. (2012). Chloroplast-mediated activation of plant immune signalling in *Arabidopsis*. Nat. Commun. 3:926. 10.1038/ncomms1926 22735454

[B94] Ott P. G. Szabó L. Balázs E. Klement Z. (1997). Submicroscopic evidence of bacterially induced resistance in tobacco leaves. Acta Phytopathol. Entomol. Hung. 32, 265–280.

[B95] Ott P. G. Varga G. J. Szatmári A. Bozsó Z. Klement E. Medzihradszky K. F.. (2006). Novel extracellular chitinases rapidly and specifically induced by general bacterial elicitors and suppressed by virulent bacteria as a marker of early basal resistance in tobacco. Mol. Plant Microbe Interact. 19, 161–172. 10.1094/MPMI-19-0161 16529378

[B96] Passardi F. Cosio C. Penel C. Dunand C. (2005). Peroxidases have more functions than a Swiss army knife. Plant Cell Rep. 24, 255–265. 10.1007/s00299-005-0972-6 15856234

[B97] Pauwels L. Goossens A. (2011). The JAZ proteins: a crucial interface in the jasmonate signaling cascade. Plant Cell 23, 3089–3100. 10.1105/tpc.111.089300 21963667 PMC3203442

[B98] Pitzschke A. Schikora A. Hirt H. (2009). MAPK cascade signalling networks in plant defence. Curr. Opin. Plant Biol. 12, 421–426. 10.1016/j.pbi.2009.06.008 19608449

[B99] Pleskot R. Pejchar P. Staiger C. J. Potockı M. (2014). When fat is not bad: the regulation of actin dynamics by phospholipid signaling molecules. Front. Plant Sci. 5:5. 10.3389/fpls.2014.00005 24478785 PMC3899574

[B100] Ranf S. Eschen-Lippold L. Pecher P. Lee J. Scheel D. (2011). Interplay between calcium signalling and early signalling elements during defence responses to microbe- or damage-associated molecular patterns. Plant J. 68, 100–113. 10.1111/j.1365-313X.2011.04671.x 21668535

[B101] Rensink W. A. Iobst S. Hart A. Stegalkina S. Liu J. Buell C. R. (2005a). Gene expression profiling of potato responses to cold, heat, and salt stress. Funct. Integr. Genomics. 5, 201–207. 10.1007/s10142-005-0141-6 15856349

[B102] Rensink W. A. Lee Y. Liu J. Iobst S. Ouyang S. Buell C. R. (2005b). Comparative analyses of six solanaceous transcriptomes reveal a high degree of sequence conservation and species-specific transcripts. BMC Genomics. 6:124. 10.1186/1471-2164-6-124 16162286 PMC1249569

[B103] Robatzek S. Chinchilla D. Boller T. (2006). Ligand-induced endocytosis of the pattern recognition receptor FLS2 in *Arabidopsis*. Genes Dev. 20, 537–542. 10.1101/gad.366506 16510871 PMC1410809

[B104] Rodríguez-Concepción M. Boronat A. (2015). Breaking new ground in the regulation of the early steps of plant isoprenoid biosynthesis. Curr. Opin. Plant Biol. 25, 17–22. 10.1016/j.pbi.2015.04.001 25909859

[B105] Rosli H. G. Zheng Y. Pombo M. A. Zhong S. Bombarely A. Fei Z.. (2013). Transcriptomics-based screen for genes induced by flagellin and repressed by pathogen effectors identifies a cell wall-associated kinase involved in plant immunity. Genome Biol. 14:R139. 10.1186/gb-2013-14-12-r139 24359686 PMC4053735

[B106] Rotter A. Usadel B. Baebler S. Stitt M. Gruden K. (2007). Adaptation of the MapMan ontology to biotic stress responses: application in solanaceous species. Plant Methods 3:10. 10.1186/1746-4811-3-10 17784939 PMC2018691

[B107] Rüegg U. T. Burgess G. M. (1989). Staurosporine, K-252 and UCN-01: potent but nonspecific inhibitors of protein kinases. Trends Pharmacol. Sci. 10, 218–220. 10.1016/0165-6147(89)90263-0 2672462

[B108] Sang Y. Cui D. Wang X. (2001). Phospholipase D and phosphatidic acid-mediated generation of superoxide in *Arabidopsis*. Plant Physiol. 126, 1449–1458. 10.1104/pp.126.4.1449 11500544 PMC117145

[B109] Santner A. Estelle M. (2010). The ubiquitin-proteasome system regulates plant hormone signaling. Plant J. 61, 1029–1240. 10.1111/j.1365-313X.2010.04112.x 20409276 PMC3066055

[B110] Segonzac C. Feike D. Gimenez-Ibanez S. Hann D. R. Zipfel C. Rathjen J. P. (2011). Hierarchy and roles of pathogen-associated molecular pattern-induced responses in *Nicotiana benthamiana*. Plant Physiol. 156, 687–699. 10.1104/pp.110.171249 21478366 PMC3177268

[B111] Segonzac C. Macho A. P. Sanmartín M. Ntoukakis V. Sánchez-Serrano J. J. Zipfel C. (2014). Negative control of BAK1 by protein phosphatase 2A during plant innate immunity. EMBO J. 33, 2069–2079. 10.15252/embj.201488698 25085430 PMC4195773

[B112] Senthil G. Liu H. Puram V. G. Clark A. Stromberg A. Goodin M. M. (2005). Specific and common changes in *Nicotiana benthamiana* gene expression in response to infection by enveloped viruses. J. Gen. Virol. 86, 2615–2625. 10.1099/vir.0.81043-0 16099921

[B113] Seo J. Lee H. Y. Choi H. Choi Y. Lee Y. Kim Y. W.. (2008). Phospholipase A_2_β mediates light-induced stomatal opening in *Arabidopsis*. J. Exp. Bot. 59, 3587–3594. 10.1093/jxb/ern208 18725378 PMC2561155

[B114] Sierro N. Battey J. N. Ouadi S. Bakaher N. Bovet L. Willig A.. (2014). The tobacco genome sequence and its comparison with those of tomato and potato. Nat. Commun. 5:3833. 10.1038/ncomms4833 24807620 PMC4024737

[B115] Spoel S. H. Mou Z. Tada Y. Spivey N. W. Genschik P. Dong X. (2009). Proteasome-mediated turnover of the transcription coactivator NPR1 plays dual roles in regulating plant immunity. Cell 137, 860–872. 10.1016/j.cell.2009.03.038 19490895 PMC2704463

[B116] Szatmári Á. Ott P. G. Varga G. J. Besenyei E. Czelleng A. Klement Z.. (2006). Characterisation of basal resistance (BR) by expression patterns of newly isolated representative genes in tobacco. Plant Cell Rep. 25, 728–740. 10.1007/s00299-005-0110-5 16456648

[B117] Szatmári Á. Zvara Á. Móricz Á. M. Besenyei E. Szabó E. Ott P. G.. (2014). Pattern triggered immunity (PTI) in tobacco: isolation of activated genes suggests role of the phenylpropanoid pathway in inhibition of bacterial pathogens. PLoS ONE 9:e102869. 10.1371/journal.pone.0102869 25101956 PMC4125134

[B118] Tao Y. Xie Z. Chen W. Glazebrook J. Chang H. S. Han B.. (2003). Quantitative nature of *Arabidopsis* responses during compatible and incompatible interactions with the bacterial pathogen *Pseudomonas syringae*. Plant Cell 15, 317–330. 10.1105/tpc.007591 12566575 PMC141204

[B119] Tena G. Boudsocq M. Sheen J. (2011). Protein kinase signaling networks in plant innate immunity. Curr. Opin. Plant Biol. 14, 519–529. 10.1016/j.pbi.2011.05.006 21704551 PMC3191242

[B120] Testerink C. Dekker H. L. Lim Z. Y. Johns M. K. Holmes A. B. Koster C. G.. (2004). Isolation and identification of phosphatidic acid targets from plants. Plant J. 39, 527–536. 10.1111/j.1365-313X.2004.02152.x 15272872

[B121] Testerink C. Larsen P. B. van der Does D. van Himbergen J. A. Munnik T. (2007). Phosphatidic acid binds to and inhibits the activity of *Arabidopsis* CTR1. J. Exp. Bot. 58, 3905–3914. 10.1093/jxb/erm243 18000017

[B122] Thilmony R. Underwood W. He S. Y. (2006). Genome-wide transcriptional analysis of the *Arabidopsis thaliana* interaction with the plant pathogen *Pseudomonas syringae* pv. *tomato* DC3000 and the human pathogen *Escherichia coli* O157:H7. Plant J. 46, 34–53. 10.1111/j.1365-313X.2006.02725.x 16553894

[B123] Trotta A. Rahikainen M. Konert G. Finazzi G. Kangasjärvi S. (2014). Signalling crosstalk in light stress and immune reactions in plants. Philos. Trans. R. Soc. Lond. B. Biol. Sci. 369:20130235. 10.1098/rstb.2013.0235 24591720 PMC3949398

[B124] Truman W. de Zabala M. T. Grant M. (2006). Type III effectors orchestrate a complex interplay between transcriptional networks to modify basal defence responses during pathogenesis and resistance. Plant J. 46, 14–33. 10.1111/j.1365-313X.2006.02672.x 16553893

[B125] Tsuda K. Sato M. Glazebrook J. Cohen J. D. Katagiri F. (2008). Interplay between MAMP-triggered and SA-mediated defense responses. Plant J. 53, 763–775. 10.1111/j.1365-313X.2007.03369.x 18005228

[B126] Tsuda K. Sato M. Stoddard T. Glazebrook J. Katagiri F. (2009). Network properties of robust immunity in plants. PLoS Genet. 5:e1000772. 10.1371/journal.pgen.1000772 20011122 PMC2782137

[B127] Usadel B. Nagel A. Thimm O. Redestig H. Blaesing O. E. Palacios-Rojas N.. (2005). Extension of the visualization tool MapMan to allow statistical analysis of arrays, display of corresponding genes, and comparison with known responses. Plant Physiol. 138, 1195–1204. 10.1104/pp.105.060459 16009995 PMC1176394

[B128] Üstün S. Börnke F. (2014). Interactions of *Xanthomonas* type-III effector proteins with the plant ubiquitin and ubiquitin-like pathways. Front. Plant Sci. 5:736. 10.3389/fpls.2014.00736 25566304 PMC4270169

[B129] Van der Luit A. H. Piatti T. van Doorn A. Musgrave A. Felix G. Boller T.. (2000). Elicitation of suspension-cultured tomato cells triggers the formation of phosphatidic acid and diacylglycerol pyrophosphate. Plant Physiol. 123, 1507–1516. 10.1104/pp.123.4.1507 10938366 PMC59106

[B130] Viehweger K. Dordschbal B. Roos W. (2002). Elicitor-activated phospholipase A_2_ generates lysophosphatidylcholines that mobilize the vacuolar H^+^ pool for pH signaling via the activation of Na^+^-dependent proton fluxes. Plant Cell 14, 1509–1525. 10.1105/tpc.002329 12119371 PMC150703

[B131] Vinitsky A. Michaud C. Powers J. C. Orlowski M. (1992). Inhibition of the chymotrypsin-like activity of the pituitary multicatalytic proteinase complex. Biochemistry 31, 9421–9428. 10.1021/bi00154a014 1356435

[B132] Vishwanath B. S. Kini R. M. Gowda T. V. (1987). Characterization of three edema-inducing phospholipase A2 enzymes from habu (Trimeresurus flavoviridis) venom and their interaction with the alkaloid aristolochic acid. Toxicon 25, 501–515. 10.1016/0041-0101(87)90286-8 3617087

[B133] Wang X. (2004). Lipid signaling. Curr. Opin. Plant Biol. 7, 329–336. 10.1016/j.pbi.2004.03.012 15134755

[B134] Wang Y. S. Pi L. Y. Chen X. Chakrabarty P. K. Jiang J. De Leon A. L.. (2006). Rice XA21 binding protein 3 is a ubiquitin ligase required for full Xa21-mediated disease resistance. Plant Cell 18, 3635–3646. 10.1105/tpc.106.046730 17172358 PMC1785399

[B135] Watt S. A. Tellstrom V. Patschkowski T. Niehaus K. (2006). Identification of the bacterial superoxide dismutase (SodM) as plant-inducible elicitor of an oxidative burst reaction in tobacco cell suspension cultures. J. Biotechnol. 126, 78–86. 10.1016/j.jbiotec.2006.02.022 16603270

[B136] Wu S. Shan L. He P. (2014). Microbial signature-triggered plant defense responses and early signaling mechanisms. Plant Sci. 228, 118–126. 10.1016/j.plantsci.2014.03.001 25438792 PMC4254448

[B137] Yan S. Dong X. (2014). Perception of the plant immune signal salicylic acid. Curr. Opin. Plant Biol. 20, 64–68. 10.1016/j.pbi.2014.04.006 24840293 PMC4143455

[B138] Yang B. Sugio A. White F. F. (2006). Os8N3 is a host disease-susceptibility gene for bacterial blight of rice. Proc. Natl. Acad. Sci. U.S.A. 103, 10503–10508. 10.1073/pnas.0604088103 16798873 PMC1502487

[B139] Yoon H. J. Kim H. K. Ma C. J. Huh H. (2000). Induced accumulation of triterpenoids in *Scutellaria baicalensis* suspension cultures using a yeast elicitor. Biotechnol. Lett. 22, 1071–1075. 10.1023/A:1005610400511

[B140] Zhu H. Li G. J. Ding L. Cui X. Berg H. Assmann S. M.. (2009). *Arabidopsis* extra large G-protein 2 (XLG2) interacts with the Gβ subunit of heterotrimeric G protein and functions in disease resistance. Mol. Plant 2, 513–525. 10.1093/mp/ssp001 19825634 PMC2902900

