# Supplementary material for: Overlapping Yet Response-Specific Transcriptome Alterations Characterize the Nature of Tobacco–Pseudomonas syringae Interactions
Source: Front Plant Sci. 2016 Mar 7;7:251. doi: 10.3389/fpls.2016.00251 (PMC4779890; doi:10.3389/fpls.2016.00251)
Supplement: Supplementary file 3 [file Table3.PDF]

Array ID[a]	N. tabacum GenBank ID[b]	Function	Forward primer	Reverse primer	microarray[c]	RT-PCR[d]	Reference[e]
STMCC79	X74453.1	Catechol O-methyltransferase	5' ATGATTGGAGCGACGAGCATT 3'	5' GCCTCTGGAAGTATGCACTC 3'	2.6[f]	3.8	1
STMER52	AJ937852.1	Glutathione S-transferase	5' GGATGGAAGTAGAATCAACAAA 3'	5' TTTCTTCATTCTCTGTAACTGC 3'	3.3	4.0	1
STMIF60	HO059472.1	NtEIG-E80 protein	5' AGCAGAGCTTAGAGGTAAACT 3'	5' GCAAGACATCAGAGGTTATCGT 3'	3.0	5.1	1
STMGQ39	X78269.1	Phenylalanine ammonia-lyase	5' AGCAACACAACCAAGATGTGAACTC 3'	5' GCTTTAAGATGTCCACGGCTTCAG 3'	2.3	3.1	1
STMCS41	DQ350353.1	Cinnamic acid 4-hydroxylase	5' TAAAACGCTTTGAACGAAGTCTA 3'	5' TTAATTGGTCTCTTCTTTGCCAT 3'	2.6	2.7	1
STMIM29	U50845.1	4-coumarate--CoA ligase	5' CGAGGCCACAACGAGAACAATAGA 3'	5' TGATCAATTCCTTCAATCGATCCA 3'	2.2	2.5	1
STMFB72	AB027753.1	peroxidase	5' ACAAAGGGTCTCAACACTCAAGATC 3'	5' GCAACCTGCGGTTCCAATA 3'	3.0	3.6	1
STMIO76	U57350.1	Epoxide hydrolase	5' CAGATGATAGCAGTAGCGGAT 3'	5' TGAATGTCGTCTTCTCGGGTT 3'	1.8	3.4	2
STMCG40	FS386581.1	peptidyl-prolyl cis-trans isomerase	5' GTATGCGAGGACTGCTATGGA 3'	5' CCCAACATAACCAACGAGGAT 3'	-2.7	-1.3	This work
STMCL93	HF564631.1	catalase	5' TCCTCTTCGACGATATTGGTATT 3'	5' CCAGTGGAACTTCACATAGGTTG 3'	-2.1	-2.9	This work
STMCP95	EB429233.1	serine-glyoxylate aminotransferase	5' CCTGCTCTCTTCCTTGTCGAT 3'	5' AAGTGCTTTCTGCGAACCAGT 3'	-3.7	-3.5	This work
STMCX90	DV161338.1	serine carboxypeptidase	5' TTGGCTTAATGGAGGTCCAGG 3'	5' ACACATTGGCAGCACGATTC 3'	-1.9	-1.5	This work
STMHE26	EB441449.1	cation proton exchanger	5' GATCTTACCCTTGTTCGGAAAGT 3'	5' GTAATGAGCAAGAATGGCGAAAG 3'	-4.9	-5.6	This work
STMHV95	KJ808744.1	remorin	5' GGCAGAAGTAGAAGCTACGAAAGT 3'	5' TTTAGGAGTTTCAACAACACGTTC 3'	-2.8	-0.9	This work

[a] EST identifier of NCBI EST database (http://www.ncbi.nlm.nih.gov/nucest/)

[b] Best *N. tabacum* homologs of potato ESTs identified by BLASTN search (http://blast.ncbi.nlm.nih.gov/Blast.cgi)

[C] Gene expression results obtained by TIGR Potato 10K microarray. *P. syringae* 61 *hrcC* induced transcription level were compared to water-treated controls at 6 hpi in tobacco leaves. Values are averages of three independent biological replicates.

[d] Gene expression results obtained by quantitative RT-PCR. *P. syringae* 61 *hrcC* induced transcription level were compared to water-treated controls at 6 hpi in tobacco leaves. Values are averages of three independent biological replicates.

[e] References of primers and RT-PCR results. 1) Szatmári, et al. (2014). Pattern triggered immunity (PTI) in tobacco: isolation of activated genes suggests role of the phenylpropanoid pathway in inhibition of bacterial pathogens. PLoS One. 7;9(8):e102869. doi: 10.1371/journal.pone.0102869; Supporting Information S3 2) Szatmári, et al. (2006). Characterisation of basal resistance (BR) by expression patterns of newly isolated representative genes in tobacco. Plant Cell Rep. 25, 728-740.

[f] gene expression in log_2 transformed form.
