# Supplementary material for: Overlapping Yet Response-Specific Transcriptome Alterations Characterize the Nature of Tobacco–Pseudomonas syringae Interactions
Source: Front Plant Sci. 2016 Mar 7;7:251. doi: 10.3389/fpls.2016.00251 (PMC4779890; doi:10.3389/fpls.2016.00251)
Supplement: Supplementary file 4 [file Table4.PDF]

Table S4. Expression of phenylpropanoid and phenolics synthesis-related genes during ETI and PTI response in tobacco leaves at 6 and 48 hpi. *P. syringae* 61 *hrcC* mutant and *P. syringae* 61 were infiltrated into leaves to induce PTI and ETI, respectively. Red and green colors represent up- or down-regulated genes, respectively. The data derived from MAPMAN metabolism overview figure, adapted for *Solanaceous* plants (Rotter et al. 2007).

id[a]	Fold-change[b] ETI 6 hpi	PTI 6 hpi	PTI 48 hpi	Similarity, Function
STMJO36	3.36	3.47		Catechol O-methyltransferase
STMHZ50	3.24	2.74		Caffeoyl-CoA O-methyltransferase
STMIC76	3.12	3.23		Cytochrome P450
STMIY78	2.97	2.53		4-coumarate-CoA ligase-like
STMEC84	2.93	3.34		Catechol O-methyltransferase
STMJM47	2.76	1.71		oxidoreductase
STMIC60	2.71	2.78	1.60	Caffeoyl-CoA O-methyltransferase
STMJE63	2.68	2.00		Tyramine hydroxycinnamoyl transferase
STMIL92	2.62	2.74		P-coumaroyl shikimate 3'-hydroxylase
STMES07	2.52	2.35		Hydroxycinnamoyl transferase
STMEI69	2.51	2.96		Catechol O-methyltransferase
STMEZ84	2.50	1.65		N-hydroxycinnamoyl-CoA:tyramine N-hydroxycinnamoyl transferase
STMJL34	2.47			oxidoreductase
STMEC76	2.42	2.21		Caffeic acid O-methyltransferase
STMGT03	2.41	2.26	1.32	Phenylalanine ammonia-lyase
STMFB08	2.41	2.14		4-coumarate-CoA ligase-like
STMGQ39	2.29	2.28	1.34	Phenylalanine ammonia-lyase
STMJL95	2.21	3.12		Caffeoyl-CoA O-methyltransferase
STMCC79	2.21	2.57		Catechol O-methyltransferase
STMIM29	2.14	2.23		4-coumarate-CoA ligase
STMGL83	2.07			cytochrome P450
STMCS41		2.64		Cinnamic acid 4-hydroxylase
STMIX91			1.58	Cinnamyl-alcohol dehydrogenase
STMGA58	-2.14	-2.12		trans-resveratrol di-O-methyltransferase-like

[a]EST identifier of NCBI EST database (http://www.ncbi.nlm.nih.gov/nucest/)

[b] gene expression in log_2 transformed form
