# Supplementary material for: Overlapping Yet Response-Specific Transcriptome Alterations Characterize the Nature of Tobacco–Pseudomonas syringae Interactions
Source: Front Plant Sci. 2016 Mar 7;7:251. doi: 10.3389/fpls.2016.00251 (PMC4779890; doi:10.3389/fpls.2016.00251)
Supplement: Supplementary file 5 [file Table5.PDF]

Table S5. Expression of redox/antioxidant- related genes during ETI and PTI response in tobacco leaves at 6 hpi and 48 hpi. *P. syringae* 61 *hrcC* and *P. syringae* 61 were infiltrated into leaves to induce PTI and ETI, respectively. Red and green colors represent up- or down-regulated genes, respectively. The data derived from MAPMAN biotic stress figure adapted for *Solanaceous* plants (Rotter et al. 2007).

id[a]	Fold-change[b]			Similarity, Function
	ETI 6 hpi	PTI 6 hpi	PTI 48 hpi	
STMER81	3.08	2.34	1.18	ascorbate oxidase
STMID82	2.85	3.61		non-symbiotic hemoglobin
STMIC34	2.79	2.13	1.76	Glutaredoxin
STMEV48	2.63	1.50		Glutaredoxin
STMCA93	-2.06	-1.85		Epimerase (ascorbate biosynthesis)
STMCL93	-2.15	-2.05		Catalase
STMDT29	-2.56	-2.31		Epimerase
STMEB83	-2.65	-2.81		Catalase
STMHX78	2.70			Glutaredoxin
STMDT51		1.73		Protein disulfide isomerase-like
STMCN77		1.72		Monodehydroascorbate reductase (ascorbate regeneration)
STMCK37		1.70		Protein disulfide isomerase-like
STMEW20		1.69		Monodehydroascorbate reductase
STMJN61		1.61		Protein disulfide isomerase
STMDP29		1.57		Protein disulfide isomerase
STMHQ72		-1.76		NADPH thioredoxin reductase
STMCV74		-2.28		Epimerase

[a]EST identifier of NCBI EST database (http://www.ncbi.nlm.nih.gov/nucest/)

[b] gene expression in $\log_2$ transformed form
