# Supplementary material for: Overlapping Yet Response-Specific Transcriptome Alterations Characterize the Nature of Tobacco–Pseudomonas syringae Interactions
Source: Front Plant Sci. 2016 Mar 7;7:251. doi: 10.3389/fpls.2016.00251 (PMC4779890; doi:10.3389/fpls.2016.00251)
Supplement: Supplementary file 13 [file Table13.PDF]

Table S13. Effect of protein kinase inhibitor K252a on selected PTI-related genes at 6 hpi. Kinase inhibitor was co-inoculated with PTI inducer *P. syringe hrcC* bacteria and gene transcription changes were compared with transcription levels induced by PTI-triggering *P. syringe hrcC* alone. Figure shows that the kinase inhibitor reduced expression of redox state, detoxification-related glutathione S-transferase, signal-related and heat-shock genes but enhanced transcription of genes involved in secondary metabolism. Gene classification was based on MAPMAN classification (Rotter et al. 2007). Red and green colors represent up- or down-regulated genes, respectively.

id[a]	Fold-change[b]	Similarity, Function
**Redox state**		
STMCK37	-1.09	Protein disulfide isomerase-like, Thioredoxin
STMDT51	-1.15	Protein disulfide isomerase-like, Thioredoxin
STMJN61	-1.74	Protein disulfide isomerase-like, Thioredoxin
STMEB83	-1.12	Catalase
**Peroxidase**		
STMHW72	-1.36	Peroxidase precursor
**Glutathione S-transferase**		
STMER52	-1.64	Glutathione S-transferase
STMET22	-1.12	Glutathione S-transferase
STMHX62	-1.09	Glutathione S-transferase
STMIK62	-1.25	Glutathione S-transferase
**Signaling**		
STMIK18	-1.47	protein kinase
STMIR02	-1.03	receptor-like serine/threonine kinase
STMIX06	-1.06	receptor protein kinase PERK1-like protein
STMIT56	-0.97	wall-associated kinase 1
**Heat shock proteins**		
STMCP23	-1.84	heat shock protein 90
STMEG24	-1.84	heat shock protein 90
STMGN23	-1.09	heat shock protein 70
**Secondary metabolites**		
STMGQ39	1.51	Phenylalanine ammonia-lyase
STMEZ84	1.39	N-hydroxycinnamoyl-CoA:tyramine N-hydroxycinnamoyl transferase
STMJE63	1.51	Tyramine hydroxycinnamoyl transferase
STMIC60	1.49	Caffeoyl-CoA O-methyltransferase
STMJO36	1.54	Catechol O-methyltransferase
STMJM47	1.99	cinnamyl alcohol dehydrogenase

[a]EST identifier of NCBI EST database (http://www.ncbi.nlm.nih.gov/nucest/)

[b] gene expression in log_2 transformed form
