# Supplementary material for: Overlapping Yet Response-Specific Transcriptome Alterations Characterize the Nature of Tobacco–Pseudomonas syringae Interactions
Source: Front Plant Sci. 2016 Mar 7;7:251. doi: 10.3389/fpls.2016.00251 (PMC4779890; doi:10.3389/fpls.2016.00251)
Supplement: Supplementary file 14 [file Table14.PDF]

Table S14. PTI-activated genes at 6 hpi that expression were blocked by *P. tabaci* and repressed by different signal inhibitors

Similarity, Function	id[a]	PTI 6 hpi[b]	$LaCl_2$[c]	neomycin	aristolochic acid	K252a	MG115
NAD-malate dehydrogenase precursor	STMEK15	1.84[d]				-1.36	
receptor-like protein kinase	STMEU79	1.63		-0.88			
Major intrinsic protein (MIP) superfamily (aquaporin)	STMGI40	2.25	-0.76	-1.21	-1.85	-2.02	
Squalene epoxidase	STMGL44	1.56	-0.90	-1.43		-1.33	-1.50
LRR transmembrane protein kinase	STMIR02	2.57				-1.03	
cell wall-associated kinase	STMIT56	2.94				-0.97	
C1-like domain uncharacterized protein	STMIX36	2.71			-1.64		
uncharacterized protein	STMJG17	1.69			-1.34		
Protein kinase	STMJN12	2.66			-1.47		

[a]EST identifier of NCBI EST database (http://www.ncbi.nlm.nih.gov/nucest/)

[b] expression level of genes activated during PTI at 6 hpi (samples were infiltrated with *P. syringe hrcC* and expression levels were compared to water injected control). The transcription of these genes did not activated by compatible *P. tabaci* comparing to water injected control.

[c] different signal inhibitors that repressed PTI-related gene expression. Inhibitors was co-inoculated with PTI inducer *P. syringe hrcC* and gene transcription changes were compared with transcription levels induced by PTI-triggering *P. syringe hrcC* alone. The inhibitors were the following: $LaCl_2$**,** Ca^{2+} channel blocker; neomycin, phospholipase C and D inhibitor; aristolochic acid, phospholipase A inhibitor; K252a, kinase inhibitor; MG115, proteasome inhibitor.

[d] gene expression in log_2 transformed form
