# Supplementary material for: Overlapping Yet Response-Specific Transcriptome Alterations Characterize the Nature of Tobacco–Pseudomonas syringae Interactions
Source: Front Plant Sci. 2016 Mar 7;7:251. doi: 10.3389/fpls.2016.00251 (PMC4779890; doi:10.3389/fpls.2016.00251)
Supplement: Supplementary file 15 [file DataSheet1.PDF]

Labeling, Hybridization and Image quantification

Total RNA samples were labeled essentially as described (Hegde et al., 2000). All control samples were labeled with the Cy5 dye and the query sample with Cy3. After hybridization and, washing and spindrying, slides were scanned using an Axon 4000B scanner (Axon Instruments, Union City, CA). Both the 635nm (red, Cy5) and 532nm (green, Cy3) channels were scanned simultaneously at 100% laser power, the PMTs (photomultiplier tube) were set between 600 and 950 to balance the signal intensities over the two channels as much as possible. Slides were scanned at a resolution of 10 micron. Images were saved in a non-compressed TIFF file format for both channels.

The TIFF images were quantified using Genepix 5.1 program (Axon Instruments, Union City, CA). Both the Cy3 and Cy5 images were analyzed simultaneously. Using a GAL-file (gene array list) the grid was overlaid on the image. Initially, print blocks of the array were identified automatically by the software and adjusted manually where needed. After block alignment the features within the blocks were identified automatically by the software. The software automatically flags spots that cannot be found in one of the channels by assigning a flag value of -50. The raw intensities of the quantified image are saved in a gpr file. Using a Perl script, additional spots are flagged that do not meet the following criteria: spots containing > 30% saturated pixels in either channel, spots with a diameter < 70 µm in either channel and spots that could not be validated during the microarray production process. These spots are all assigned a flag value of -100. The background intensity is calculated by the Genepix software as follows: the median pixel intensity is calculated from a circular region with three times the diameter of the spot, excluding the pixels assigned to neighboring spots. Median background intensity is used to reduce the effect of spurious pixels contributing to the background.

For background correction and data normalization for each hybridization the raw intensities are loaded into the limma package of Bioconductor (www.bioconductor.org) using the read.maimages function. For the Cy5 channel the F635 Mean column of the gpr-file is used as foreground intensity and the B635 Median column is used as background intensity. For the Cy3 channel the F532 Mean and B532 Median columns are used as foreground and background intensities. Spots with a negative Flag value (Flags column in the gpr file) are assigned a weight of 0 using the wt.fun function of the limma package. Background subtraction and normalization

is performed by the normalizeWithArrays function of limma. Background intensities are subtracted from the foreground intensities and negative values are set to 0. Background corrected intensities are normalized by the print-tip loess method using default parameters. Because the flagged spots are assigned a weight of 0, these spots are excluded from the normalization process (and loess curve fitting). The normalized and background subtracted intensity values are exported from limma both for the red (Cy5) and green (Cy3) channel, the flagged spots are assigned a value of "NA".
