# Supplementary material for: Overlapping Yet Response-Specific Transcriptome Alterations Characterize the Nature of Tobacco–Pseudomonas syringae Interactions
Source: Front Plant Sci. 2016 Mar 7;7:251. doi: 10.3389/fpls.2016.00251 (PMC4779890; doi:10.3389/fpls.2016.00251)

Data Sheet 2. General down-regulation of photosynthesis/chloroplast related genes during PTI (**A, C**) and ETI (**B**) in tobacco leaves. *P. syringae* 61 *hrcC* and *P. syringae* 61 were infiltrated into leaves to induce PTI and ETI, respectively. Red and green colors represent up- or down-regulated genes, respectively. The figures were derived from MAPMAN software adapted for *Solanaceous* plants (Rotter et al. 2007).

A

PTI 6 hpi

B

ETI 6 hpi

C

ETI 48 hpi

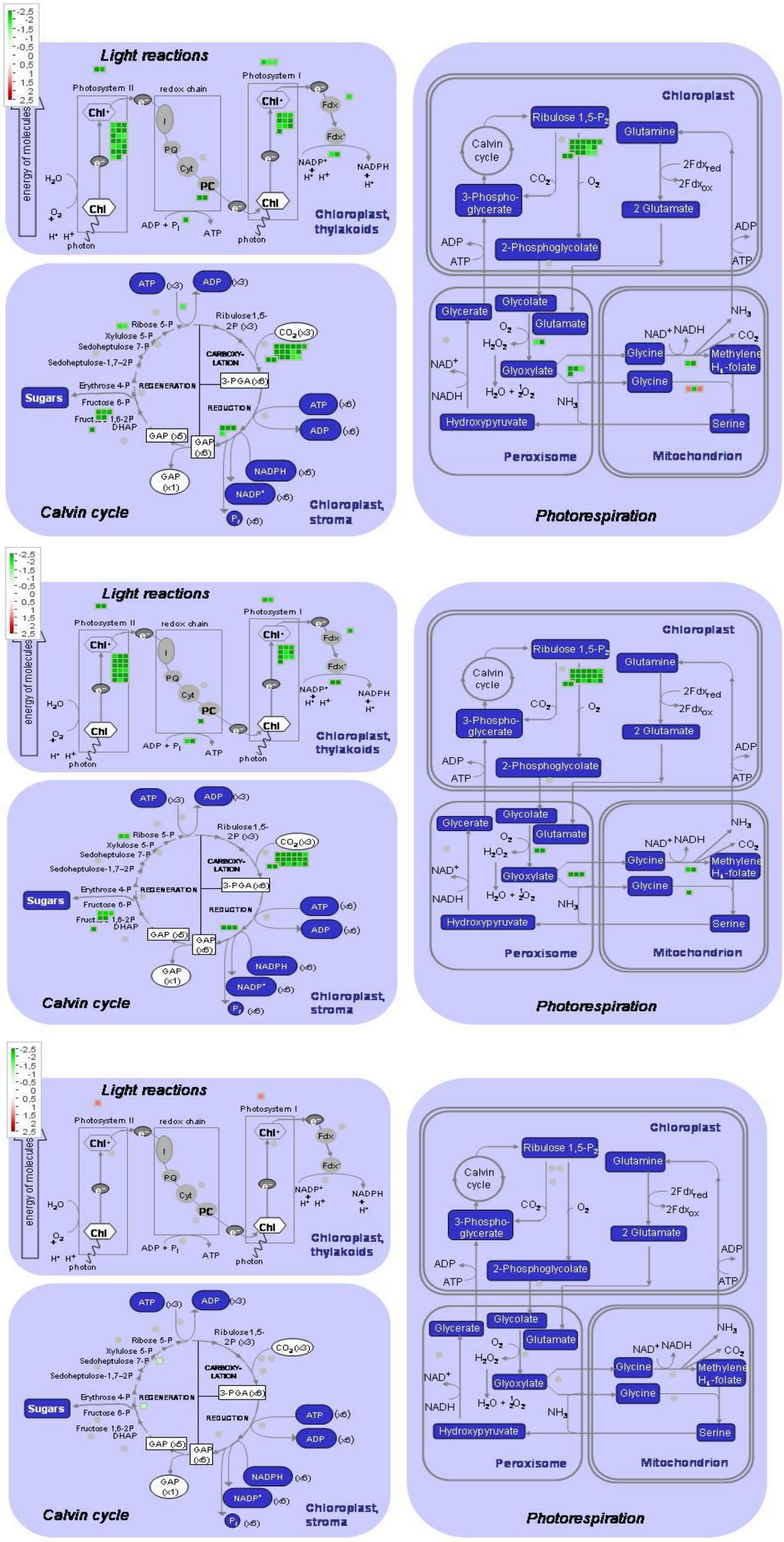

Supplement: Supplementary file 16 [file DataSheet2.pdf]
