# Supplementary material for: Overlapping Yet Response-Specific Transcriptome Alterations Characterize the Nature of Tobacco–Pseudomonas syringae Interactions
Source: Front Plant Sci. 2016 Mar 7;7:251. doi: 10.3389/fpls.2016.00251 (PMC4779890; doi:10.3389/fpls.2016.00251)

Data Sheet 3. A simplified model of possible signaling pathways involved in regulation of PTI-related gene expressions. Only those pathway elements are presented here that were inhibited by various pharmacological agents in this study. The used inhibitors are in red circles (neomycin is connected to PLD with dashed line because it inhibits only phosphatidylcholine-specific PLD activity). Thicker arrows show more pronaunced interactions and pathways suggested by microarray results in this study. Not all possible interactions are indicated in figure. Question mark between PLC and proteasome indicates that the type of the interactions is unclear. Details are in the text and in Fig 5. PLA_2 phospholipase A2;PLC: phospholipase C; PLD: phospholipase D.

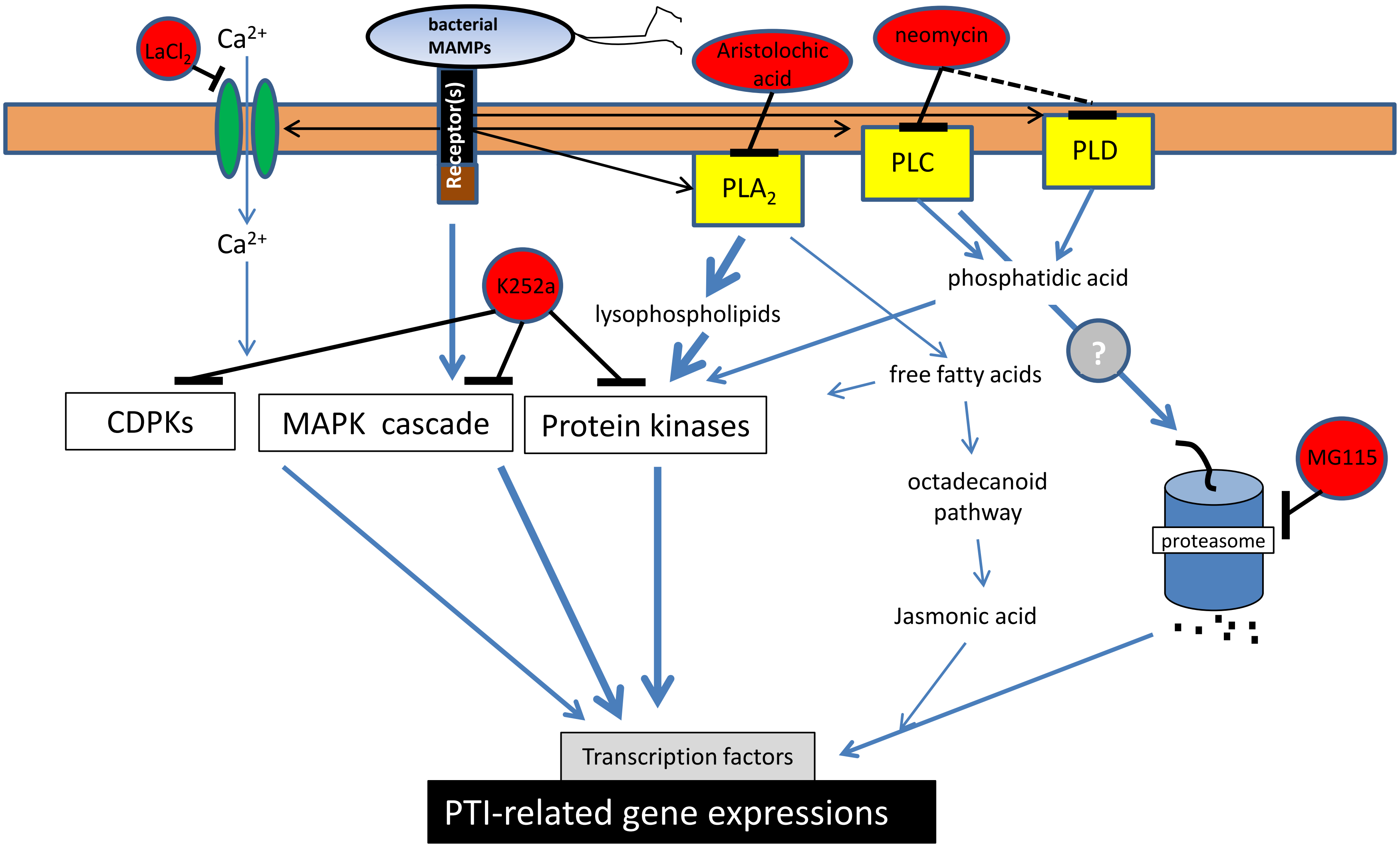

Supplement: Supplementary file 17 [file DataSheet3.pdf]
